# Identification of a primitive intestinal transcription factor network shared between esophageal adenocarcinoma and its precancerous precursor state

**DOI:** 10.1101/gr.243345.118

**Published:** 2019-05

**Authors:** Connor Rogerson, Edward Britton, Sarah Withey, Neil Hanley, Yeng S. Ang, Andrew D. Sharrocks

**Affiliations:** 1 School of Biological Sciences, Faculty of Biology, Medicine and Health, University of Manchester, Manchester M13 9PT, United Kingdom;; 2 School of Medical Sciences, Faculty of Biology, Medicine and Health, Manchester Academic Health Sciences Centre, University of Manchester, Manchester M13 9PT, United Kingdom;; 3 Endocrinology Department, Central Manchester University Hospitals NHS Foundation Trust, Manchester M13 9WU, United Kingdom;; 4 GI Science Centre, Salford Royal NHS FT, University of Manchester, Salford M6 8HD, United Kingdom

## Abstract

Esophageal adenocarcinoma (EAC) is one of the most frequent causes of cancer death, and yet compared to other common cancers, we know relatively little about the molecular composition of this tumor type. To further our understanding of this cancer, we have used open chromatin profiling to decipher the transcriptional regulatory networks that are operational in EAC. We have uncovered a transcription factor network that is usually found in primitive intestinal cells during embryonic development, centered on HNF4A and GATA6. These transcription factors work together to control the EAC transcriptome. We show that this network is activated in Barrett's esophagus, the putative precursor state to EAC, thereby providing novel molecular evidence in support of stepwise malignant transition. Furthermore, we show that HNF4A alone is sufficient to drive chromatin opening and activation of a Barrett's-like chromatin signature when expressed in normal human epithelial cells. Collectively, these data provide a new way to categorize EAC at a genome scale and implicate HNF4A activation as a potential pivotal event in its malignant transition from healthy cells.

Esophageal adenocarcinoma (EAC) is one of the eight most common cancers in the Western world and it has very low survival rates (Pennathur et al. 2013). One reason for the poor prognosis is the lack of tailored therapies due to the relative paucity of molecular knowledge compared to other cancers. We are beginning to understand the molecular mechanisms underpinning this disease, chiefly through genomic sequencing studies, which have revealed multiple genes that are recurrently mutated in EAC (Dulak et al. 2013; Weaver et al. 2014; Ross-Innes et al. 2015; Frankell et al. 2019). However, with the exception of *TP53*, the overall incidence of mutations in individual genes is low. By grouping genes in broader functional categories, frequently mutated pathways have been uncovered, such as chromatin remodeling complexes, the RAS-ERK signaling pathway, and cell cycle control pathways. Another broader category of interest is comprised of genes encoding transcriptional regulatory proteins that include both GATA4 and GATA6. Together, amplification of the genes encoding these two transcription factors has been reported in up to 40% of EAC cases (Lin et al. 2012; The Cancer Genome Atlas Research Network 2017). This suggests that transcriptional network rewiring might be an important element in the progression of EAC.

There are persuasive arguments in favor of EAC developing from a preexisting condition known as Barrett's esophagus (Burke and Tosh 2012; Desai et al. 2012). EAC and Barrett's share a large number of molecular markers. For example, many of the mutations found in EAC are already present in Barrett's, including in *TP53*, suggesting that they may help drive the transition to Barrett's as a stepwise process to EAC, rather than to EAC directly from healthy tissue (Ross-Innes et al. 2015; Stachler et al. 2015). In contrast, focal gene amplifications tend to arise following the transition from Barrett's to EAC, suggesting that these may play a more important role in establishing the EAC state (Lin et al. 2012; Ross-Innes et al. 2015; Yamamoto et al. 2016). Morphologically, the normal esophagus consists of a stratified squamous epithelium, but Barrett's differs significantly from this and instead resembles a columnar epithelium, typically found in the more distal gastrointestinal tract (for review, see Spechler and Souza 2014). Several models have been proposed for how this metaplastic transition occurs including transdifferentiation from normal esophageal epithelial cells (Stairs et al. 2008; Vega et al. 2014), colonization by migrating cells of gastric origin (Quante et al. 2012), changes to cells from the gastroesophageal junction (GOJ) (Jiang et al. 2017), or more recently, from cellular subpopulations within normal esophageal epithelia (Owen et al. 2018).

Despite these advances, mutational signatures have not yet provided a unified insight into how EAC is initiated and maintained. Changes to the epigenetic landscape might be a major contributing factor, and this scenario has been implicated in other cancers (Davie et al. 2015; Rendeiro et al. 2016). To gain insights into the molecular mechanisms that are operational in EAC, we therefore turned to ATAC-seq to study the open chromatin landscape as this has been successfully applied to studying cell fate transitions such as neuron differentiation from fibroblasts (Wapinski et al. 2017), hematopoiesis (Yu et al. 2016), epidermal differentiation (Bao et al. 2015), and embryonic development (Cusanovich et al. 2018). In the current study, we interrogated the chromatin landscape of patient samples to discover the regulatory networks operational in Barrett's esophagous and EAC.

## Results

### Identification of a network of transcription factors active in EAC

Previously we used ATAC-seq to profile the open chromatin landscape of EAC cell lines and identified AP-1 as an important transcription factor family in controlling the transcriptional networks in EAC (Britton et al. 2017). To further interrogate the transcriptional networks operating in EAC, we decided to take an alternative approach, starting with clearly defined open chromatin data sets from EAC patient samples rather than a diverse set of EAC-derived cell lines. We previously validated our cell line–derived results by profiling the open chromatin of six patient-derived biopsies, but this revealed two distinct subclusters of EAC samples based on their open chromatin landscapes: one that clustered with normal samples and one that was unique (Britton et al. 2017). We revisited this issue by performing subclustering by PCA analysis following the addition of an additional paired normal and EAC data set and again we observed a clear partitioning of samples (Supplemental Fig. S1A). We therefore refocused our attention on the four EAC samples that are clearly distinct from the normal samples (T_002, T_003, T_005, T_006) (Supplemental Fig. S1A).

**Figure 1.**
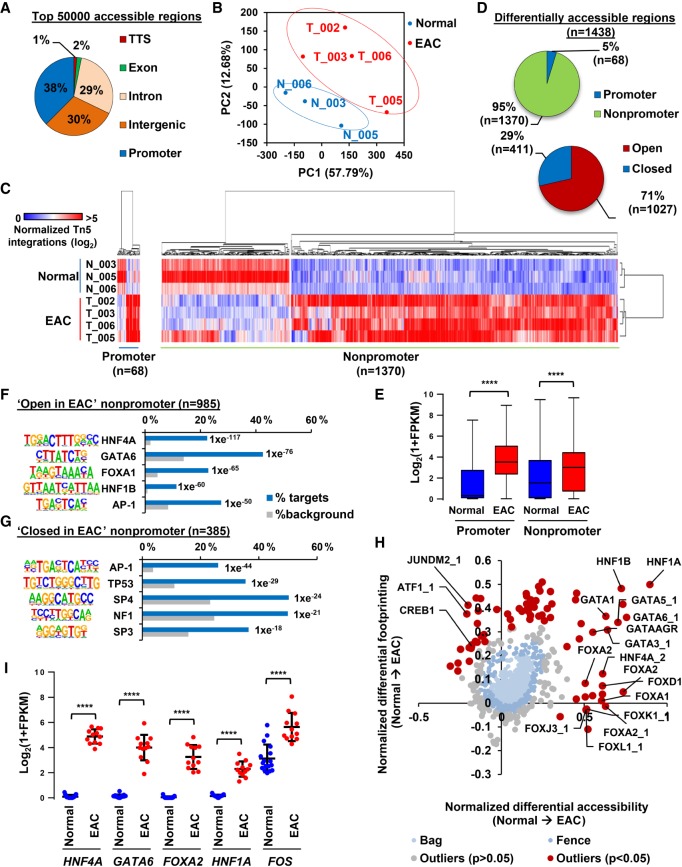
Open chromatin profiling reveals dynamic chromatin accessibility in EAC. (*A*) Genomic distribution of the top 50,000 significant open chromatin regions in combined ATAC-seq data from normal and tumor tissue. “Promoter” refers to −2.5 to +0.5 kb relative to the TSS. (*B*) PCA plot of ATAC-seq signal across the top 50,000 ATAC-seq regions in three normal tissue samples (blue) and four tumor tissue samples (red). (*C*) Heatmap of normalized Tn5 cleavage events in a ±250-bp region surrounding the summits of differentially accessible promoter and nonpromoter regions (linear fivefold difference, *Q* < 0.05). Hierarchical clustering was performed on samples and regions using 1-Pearson's correlation. (*D*) Pie charts representing the proportion of differentially accessible regions that are located in promoter (−2 kb, +0.5 kb of TSS) and nonpromoter regions (*left*) and regions that are fivefold more open or closed in tumor tissue (*right*). (*E*) Box plot of Log_2_(1 + FPKM) values of genes associated with promoter and nonpromoter regions that show increased accessibility in EAC (red) compared to normal tissue (blue). Whiskers represent 1.5× IQR. (*F*,*G*) The top five DNA motifs derived from de novo motif discovery and their associated transcription factor that are enriched in “open in cancer” (*F*) or “closed in cancer” (*G*) nonpromoter regions. The frequency of motif occurrence is shown, and the motifs are sorted by *P*-value. (*H*) Scatter bag plot of differential chromatin accessibility (*x*-axis) and footprinting (*y*-axis) depth around human transcription factor binding motifs in normal and cancer tissue. Significant outliers (*P* < 0.05) are represented in red. Transcription factor motifs with enrichment in “open in cancer” are labeled. (*I*) Plot of Log_2_(1 + FPKM) values of transcription factors with enriched motifs in “open in cancer” regions in RNA-seq data from normal (blue) or EAC (red) tissue (Maag et al. 2017). Mean is represented by a black bar with standard deviation shown *above* and *below*. (****) *P* < 0.0001.

To derive a unified data set of open chromatin regions in esophageal-derived tissue, we combined all the ATAC-seq data from three normal esophageal tissue samples (matched with T_003, T_005, and T_006) and these four EAC samples and recalled the open accessible chromatin regions from this combined data. We selected the top 50,000 open regions (Supplemental Table S1A) and found that these were roughly equally divided between promoter-proximal (−2 kb, +0.5 kb from TSS) intragenic and intergenic regions (Fig. 1A). PCA analysis based on these peaks confirmed the distinct clustering of the normal and EAC samples (Fig. 1B). Next, we identified regions that are differentially accessible between the normal and EAC samples by comparing the average signals in each type of sample. This yielded a total of 1438 differentially accessible regions, the vast majority of which (95%) are located in intra- or intergenic regions (Fig. 1C,D; Supplemental Table S1B,C). Clustering analysis using these differentially accessible regions clearly separates the normal and EAC samples and revealed that chromatin opening is more prevalent than closing in EAC (71% of regions are more open in EAC) (Fig. 1C,D). The differentially accessible regions were linked to likely regulated genes using the nearest gene model. Subsequent GO terms analysis revealed that these open regions are associated with genes with functions such as “cell development,” “response to hormone stimulus,” and “response to wounding” (Supplemental Fig. S1B). We then asked whether opening of chromatin corresponds with an increase in gene expression. We interrogated RNA-seq data from normal esophageal and EAC tissue (Maag et al. 2017) and found that genes associated with a region demonstrating an increase in accessibility (promoter and nonpromoter) in EAC also show elevated levels of expression in EAC (Fig. 1E).

To gain further insights into the regulatory networks that act on these differentially open chromatin regions, we first identified DNA motifs that are overrepresented in the differentially accessible nonpromoter regions (Fig. 1F; Supplemental Table S2A). As expected from our previous work (Britton et al. 2017), we found AP-1 motifs in the differentially open regions in EAC but additionally we found greater enrichment of binding motifs for the HNF4, GATA, FOXA, and HNF1 subfamilies of transcription factors (Fig. 1F). Of these motifs, HNF4 was also found at promoter regions (Supplemental Fig. S1C). In contrast, with the exception of AP-1, a different set of DNA motifs are associated with regions closed in cancer, with the TP53 motif being the most prevalent (Fig. 1G). In addition to demarcating open chromatin, ATAC-seq can also reveal the precise sequence bound by a transcription factor by the bound transcription factor protecting the underlying DNA sequence from digestion (the “footprint”). For robustness, we used two complementary approaches. By the Wellington algorithm (Piper et al. 2013), we again identified the motifs for HNF4A/G and GATA6 (Supplemental Fig. S1D). Next, we used BaGFoot (Baek et al. 2017) to identify motifs that showed evidence of changes of occupancy through altered “footprinting” in the EAC samples across all the top 50,000 open regions in the tissue-derived ATAC-seq data sets. Again, we identified motifs for the HNF4, GATA, FOXA, and HNF1 transcription factor subfamilies in this analysis that all exhibited greater localized accessibility in cancer, with the GATA6 and HNF1B motifs showing particularly strong increases in footprinting depth across the motif itself (Fig. 1H; Supplemental Fig. S2). Conversely, the TP53 motif showed evidence of reduced footprinting depth and local chromatin accessibility (Supplemental Fig. S2). Given the enrichment of these TF motifs, we next determined whether any of the transcription factors which recognize these motifs are up-regulated in EAC. Again, we used RNA-seq data from normal esophageal and EAC tissue (Maag et al. 2017) and found that GATA4/6, and HNF4, FOXA, and HNF1 family members are all up-regulated in EAC samples, albeit with different subfamily members in individual patients (Fig. 1I; Supplemental Fig. S1E). Furthermore, representative genes encoding transcription factors from these subclasses also show increased ATAC-seq signal in their putative regulatory regions in EAC tissue compared to normal tissue (Supplemental Fig. S1F), consistent with their transcriptional up-regulation. To determine whether these transcription factors may form a network in EAC, we focused on HNF4A and GATA6 motifs in regions of open chromatin and counted the frequency of co-occurring motifs for HNF4A, GATA6, FOXA1, HNF1B, and AP-1 within the same regions. For both HNF4A and GATA6 motifs, there is a significantly different distribution of co-occurring motifs in regions that are more accessible in EAC compared to randomly selected genomic regions containing either of these motifs (Supplemental Fig. S1G). This is particularly marked for the co-occurrence of HNF4A with GATA6 motifs and suggests the existence of a complex transcription factor network in EAC.

Given the conflicting theories about how EAC arises, we wanted to explore where else this regulatory pattern of transcription factor activity might be observed. Given the highest enrichment for the GO term “regulation of cell development” (Supplemental Fig. S1B), we hypothesized that EAC might be mimicking aspects of human embryogenesis, and in particular distal foregut endoderm (Gerrard et al. 2016). Indeed, the cohort of nine transcription factors was most highly enriched in embryonic derivatives of distal foregut endoderm (stomach and liver) compared to proximal derivatives (thyroid or lung) or tissues mainly derived from other germ layers, for example, brain (ectoderm) or heart ventricle, adrenal, or kidney (mesoderm) (Supplemental Fig. S3). However, strong coexpression was not observed in any single tissue type. To explore this further, we integrated the human embryonic data with RNA-seq from differentiating human pluripotent stem cells. The strongest enrichment was observed at the stage of foregut endoderm/early liver differentiation, where representative members from each of the four subfamilies are coexpressed.

Collectively, these data implicate HNF4A, GATA6, FOXA2/3, and HNF1B as a group of transcription factors specific to EAC controlling gene expression predominantly through distal regulatory sites. This complement of factors points to EAC arising due to a reactivation of a distal human embryonic foregut phenotype.

### Identifying the GATA6 and HNF4A cistrome

Because GATA6 and HNF4A show the highest motif occurrence and the highest differential expression in EAC, we focused on these two transcription factors and aimed to determine their role in controlling gene expression in EAC cells. We first sought a suitable cell line whose open chromatin environment resembled that found in the patient-derived EAC samples. Initially we used PCA to cluster the open chromatin regions from four EAC-derived cell lines with the patient-derived samples. OE19 cells were clearly identified as most closely resembling the open chromatin landscape of primary EAC (Supplemental Fig. S4A). This was confirmed by Pearson's correlation analysis (Supplemental Fig. S4B). Additionally, both GATA6 and HNF4A levels are elevated in OE19 cells compared to the other cell lines (Supplemental Fig. S4C). *CLRN3* has been identified as a HNF4A transcriptional target in iPSC-derived hepatocytes (Mallanna et al. 2016), and *CLDN18* has been confirmed as a GATA6 target in gastric cancer (Sulahian et al. 2014); therefore, we used these likely targets to test the regulatory potential of HNF4A and GATA6 in OE19 cells. Both these genes exhibit multiple regions of increased chromatin accessibility in both tumor tissue and OE19 cells, several of which contain motifs for HNF4A or GATA6 binding (Supplemental Fig. S4D). ChIP-qPCR confirmed occupancy of regions by HNF4A and GATA6 containing their cognate binding motifs, whereas control regions lacking these motifs showed little evidence of binding (Supplemental Fig. S4E).

**Figure 2.**
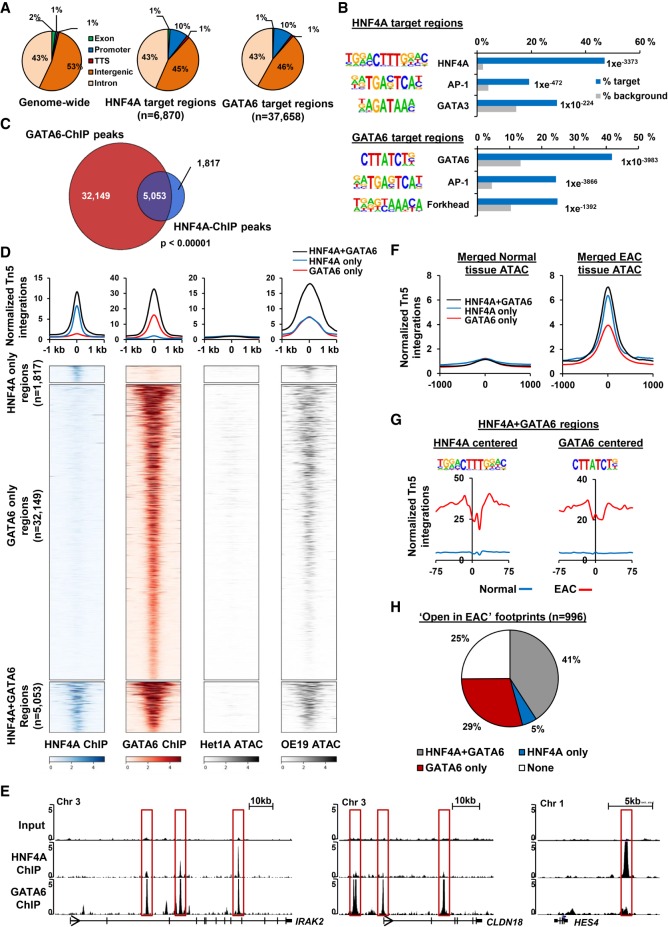
Genome-wide identification of HNF4A and GATA6 binding sites. (*A*) Genomic distribution of HNF4A and GATA6 ChIP-seq regions compared to genomic average. Promoter is defined as −2.5 to +0.5 kb relative to the TSS. (*B*) The top three motifs from de novo motif discovery and their associated transcription factor found in ChIP-seq regions for HNF4A (*top*) and GATA6 (*bottom*). The frequency of motif occurrence is shown, and motifs are sorted by *P*-value. (*C*) Venn diagram of overlapping binding regions from HNF4A and GATA6 ChIP-seq. *P*-value was calculated using Fisher's exact test. (*D*) Heatmap and tag density plots of ChIP-seq signals for HNF4A and GATA6 at regions bound by HNF4A only, GATA6 only, or both HNF4A and GATA6. The ATAC-seq signals at the same regions in Het1A and OE19 cells are shown. (*E*) UCSC Genome Browser tracks showing ChIP-seq tracks at three loci (*IRAK2*, *CLDN18*, and *HES4*). GATA6 and/or HNF4A bound regions are boxed. (*F*) Tag density plots of ATAC-seq signal at regions bound by HNF4A only, GATA6 only, or both HNF4A and GATA6 in normal and EAC tumor tissue. (*G*) Footprinting of ATAC-seq data from normal and EAC tissue (*bottom*) on 150-bp regions surrounding the HNF4A or GATA6 transcription factor motifs found in HNF4A + GATA6 co-occupied ChIP-seq regions. (*H*) Distribution of ATAC-seq footprints in “open in cancer” regions that coincide with ChIP-seq regions of HNF4A and GATA6.

Next, to gain a more comprehensive view of the role of HNF4A and GATA6, we expanded our analysis of their cistromes by ChIP-seq in OE19 cells. The HNF4A and GATA6 antibodies robustly precipitated the respective proteins (Supplemental Fig. S5A), and replicate ChIP-seq experiments were highly reproducible (Supplemental Fig. S5B,C). We therefore took the overlap of the two replicates forward for further analyses, resulting in 6870 and 37,658 binding regions for HNF4A and GATA6, respectively (Supplemental Table S3). To ensure that all peaks taken forward were functional, we ranked peaks from both data sets by enrichment, partitioned peaks into 10% bins, and searched for respective motifs. All bins showed a high enrichment of motifs ranging from 87% to 32% for HNF4A and 70% to 28% for GATA6 (Supplemental Fig. S5D), therefore we kept all peaks for further analyses. Very similar genomic distributions were observed for each transcription factor, with the majority (90%) being in intra- or intergenic regions (Fig. 2A). DNA motif analysis identified HNF4A and GATA6 as the top enriched motifs in their respective data sets (>42% of regions in both cases) (Fig. 2B), and although these motifs tended to be more prevalent in the higher confidence binding regions, these motifs were distributed throughout the entire data sets (Supplemental Fig. S5D). We identified AP-1 as the next most enriched motif in both cases, in keeping with our previous finding of AP-1 as an important factor in EAC (Britton et al. 2017); in addition, we also identified GATA motifs in the HNF4A binding regions and Forkhead binding motifs in the GATA6 binding regions (Fig. 2B; Supplemental Table S4). Although the HNF4A motif was not among the top enriched motifs in the GATA6 ChIP-seq data set, searching for the HNF4A DNA binding motif within GATA6-bound regions revealed that 8.1% of GATA6 ChIP-seq regions contain a HNF4A motif (Supplemental Fig. S5E). These observations are in keeping with our identification of the same motifs in the context of open chromatin regions in EAC (Fig. 1F) and suggest an integrated network of transcription factors. Indeed, the majority (74%) of HNF4A binding regions are also occupied by GATA6 (Fig. 2C,D). One locus showing such co-occupancy is *IRAK2*, although uniquely bound regions can be identified in other loci including *CLDN18* and *HES4* (Fig. 2E). The regions co-occupied by both HNF4A and GATA6 exhibit low levels of open chromatin in noncancerous esophageal Het1A cells, but they show elevated levels of open chromatin in OE19 cells compared to the single occupied regions, suggesting that these may have more regulatory potential (Fig. 2D).

To relate these findings back to patient-derived samples, we asked whether the HNF4A and GATA6 binding regions are accessible in our biopsies from normal tissue and EAC. Little evidence of open chromatin is apparent in normal tissue, but elevated levels are seen in EAC tissue, which is particularly marked in regions occupied by HNF4A alone and co-occupied by both HNF4A and GATA6 in OE19 cells (Fig. 2F). We also asked whether we could detect changes in DNA accessibility in and around the HNF4A and GATA6 binding motifs within their binding regions, when comparing data from normal and EAC samples. Clear footprints were observed centered on the HNF4A motifs in EAC tissue, which were particularly prominent in regions co-occupied with GATA6 (Fig. 2G; Supplemental Fig. S5F). Similarly, we also observed a footprint around the GATA6 motifs in EAC tissue, but this was only observed in the co-occupied regions (Fig. 2G; Supplemental Fig. S5F). Similar results were obtained when comparing chromatin accessibility data from cell line models (Supplemental Fig. S5F). Finally, we asked whether any of the footprints we identified in the patient-derived EAC material overlapped with the binding regions for HNF4A and GATA6 and found that 75% of these overlapped with one or other factor, with the largest percentage (41%) being associated with the regions cobound by HNF4A and GATA6 (Fig. 2H).

Collectively, these data demonstrate that HNF4A and GATA6 bind to a large number of regulatory regions in EAC cells, often showing co-occupancy. These co-occupied sites are potentially more functionally relevant in EAC because they are associated with more open chromatin and deeper footprints in both cell line models and patient samples.

### The GATA6 and HNF4A regulome

**Figure 3.**
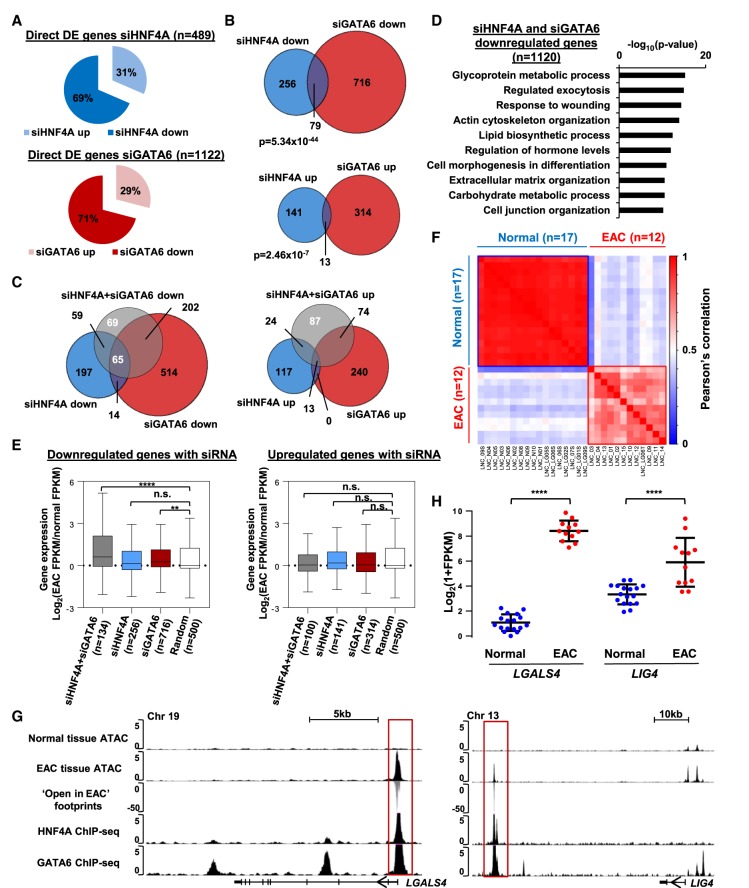
HNF4A and GATA6 target genes are overexpressed in EAC tumors. (*A*) Proportion of differentially expressed (DE) genes (>1.3-fold; *Q*-value <0.05) that are up- and down-regulated in OE19 cells following siHNF4A (*top*) and siGATA6 (*bottom*) treatment. (*B*) Overlap of differentially expressed genes in the siHNF4A and siGATA6 data sets. *P*-value calculated using the hypergeometric test. (*C*) Overlap of differentially expressed genes that are down-regulated following siHNF4A and siGATA6 cotreatment compared to single siRNA treatments. (*D*) The top 10 significant “biological processes” GO term analysis of genes down-regulated following siHNF4A, siGATA6, or siHNF4A + siGATA6 treatment. (*E*) Box plot of changes in gene expression in EAC tissue (shown as cancer/normal FPKM log_2_ fold change) for genes down-regulated and up-regulated by siRNA against HNF4A (blue), GATA6 (red), or both (gray). Whiskers represent 1.5× IQR. A random (500 randomly selected RefSeq transcripts) data set is represented in white. (****) *P* < 0.0001; (**) *P* < 0.01. (*F*) Pearson correlation plot of Log_2_(1 + FPKM) values of siHNF4A + siGATA6-regulated genes in normal (*n* = 17) and EAC (*n* = 12) patient samples (Maag et al. 2017). Samples were clustered hierarchically, and the two main clusters are highlighted as a “normal” cluster (blue) and an “EAC” cluster (red). (*G*) UCSC Genome Browser tracks showing ATAC-seq data in normal and tumor tissue (*top*), “open in cancer” footprints (*center*), and HNF4A and GATA6 ChIP-seq signals (*bottom*) at genes regulated by HNF4A and GATA6, *LGALS4* and *LIG4*. (*H*) Plot showing the expression of *LGALS4* and *LIG4* in normal (blue) and EAC (red) tissue samples (Maag et al. 2017). Mean is represented by a black bar with standard deviation shown *above* and *below*. (****) *P* < 0.0001.

Having established the HNF4A and GATA6 cistromes, we next determined their effects on gene expression by depleting them individually and in combination in OE19 cells. Efficient depletion of both transcription factors was achieved at both the RNA and protein levels (Supplemental Fig. S6A–D), and the expected down-regulation of the target genes *CLRN3* and *CLDN18* was achieved by depletion of HNF4A and GATA6, respectively (Supplemental Fig. S6B,D). RNA-seq was then performed, data quality verified (Supplemental Fig. S6E), and differentially expressed genes identified. We focused on likely direct target genes (defined as being associated with an annotated ChIP-seq peak using the nearest gene model) and identified 489 and 1122 genes whose expression was changed by >1.3-fold (*Q*-value <0.05) following HNF4A and GATA6 depletion, respectively (Fig. 3A; Supplemental Table S5A,B). As expected for activating transcription factors, the majority (∼70% in both cases) were down-regulated. Neither factor seems to be largely regulated by the other (Supplemental Fig. S6B,D). Although there is a large overlap in binding, the overlap in genes down- or up-regulated by each protein was relatively modest (albeit very significant), suggesting that either HNF4A or GATA6 might be the more dominant factor at different genes (Fig. 3B). To identify further potentially regulated genes, we performed RNA-seq following depletion of both HNF4A and GATA6. This identified a further 156 deregulated genes (69 down- and 87 up-regulated) when both factors are depleted together in addition to the 92 genes that are commonly reregulated by either treatment alone (Fig. 3C; Supplemental Table S5C). GO term analysis of all the genes down-regulated by depletion of either HNF4A or GATA6 showed enriched GO terms for “Glycoprotein metabolic process,” “Response to wounding,” and “Regulation of hormone levels” (Fig. 3D). Similar terms were uncovered when individually analyzing the genes deregulated by depletion of either factor alone (Supplemental Fig. S6F). Several of these GO terms are also similar to the GO terms identified in regions that are specifically accessible in patient-derived EAC samples (Supplemental Fig. S1C), indicating the HNF4A and GATA6 regulome contributes to the overall phenotype of EAC.

To determine whether the HNF4A- and GATA6-regulated genes are relevant to EAC, we examined whether any changes in their expression could be observed in EAC compared to normal esophageal tissue. We focused on the genes that were down-regulated following siRNA treatment because these are more likely direct targets normally activated by these transcription factors. Significantly higher expression of the genes activated by both HNF4A and GATA6 in OE19 cells was observed in EAC tissue, with a lower but still significant increase in expression for the cohort of genes activated by GATA6 alone (Fig. 3E). In contrast, the genes that are up-regulated following siRNA treatment (i.e., likely indirect effects) are not expressed at higher levels in EAC tissue (Fig. 3E). An identical trend was observed in a different data set, with highest expression in cancer being observed for genes activated by both HNF4A and GATA6 (Supplemental Fig. S7). Hierarchical clustering using Pearson's correlation of the expression of genes activated by both factors in normal and EAC tissue completely separates the two tissues (Fig. 3F), again suggesting that the expression of HNF4A, GATA6, and their target genes are biologically relevant. Two example genes from this category are *LGALS4* and *LIG4* (Fig. 3G). Both are direct targets for HNF4A and GATA6, both are associated with regions of open chromatin around these sites only in EAC tissue, and both have EAC-specific footprints. These changes in HNF4A and GATA6 binding activity are also associated with increased gene expression in the context of EAC (Fig. 3H).

Collectively, these data demonstrate that HNF4A and GATA6 directly regulate a set of genes that are expressed at higher levels in EAC tissue and are consistent with our identification of HNF4A and GATA6 binding motifs in the open regulatory regions that are specific to EAC.

### The HNF4A-GATA6 regulatory network is operational in Barrett's esophagus

**Figure 4.**
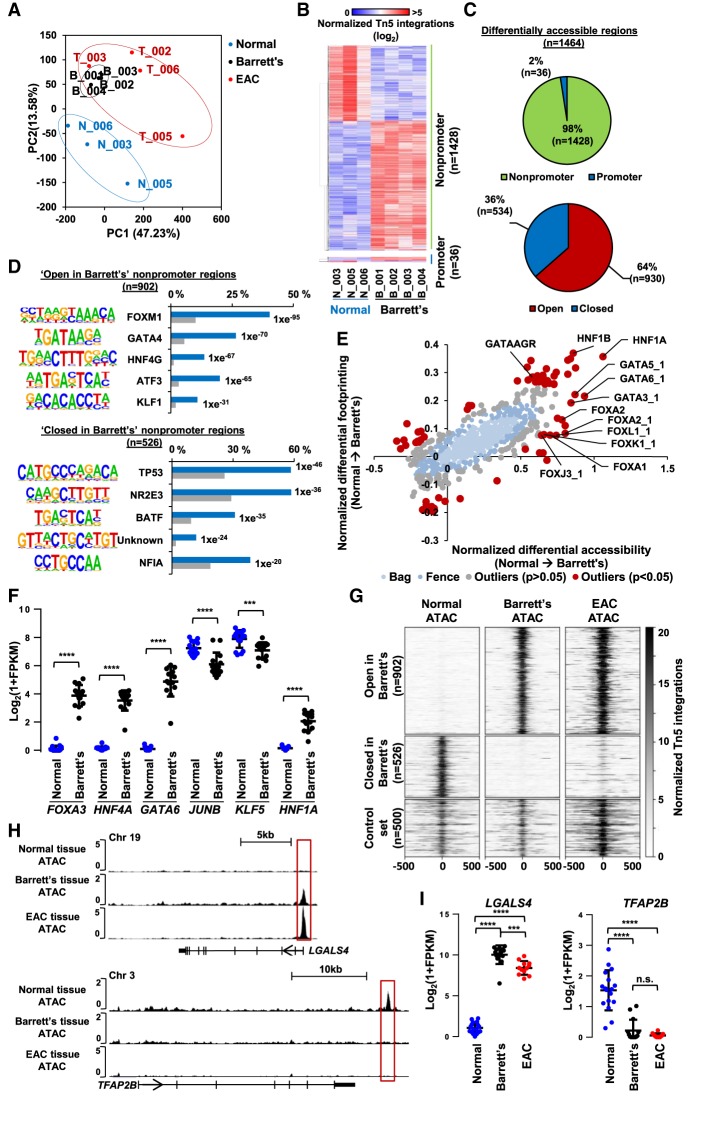
Barrett's esophagus ATAC-seq is related to EAC. (*A*) PCA plot of the normalized ATAC-seq signal across the top 50,000 regions of all three normal (blue), four Barrett's (black), and four EAC (red) tissue samples. (*B*) Heatmap of normalized Tn5 cleavage events in a ±250-bp region surrounding the summit of differentially accessible nonpromoter and promoter regions (greater than fivefold change, *Q* < 0.05) in normal versus Barrett's samples. (*C*) Pie charts representing the proportion of differentially accessible regions that are located in promoter and nonpromoter regions (*top*) and regions that are fivefold more open or closed in Barrett's tissue (*bottom*). (*D*) Top five DNA motifs derived from de novo motif discovery and their associated transcription factor that are enriched in “open in Barrett's” (*top*) or “closed in Barrett's” (*bottom*) nonpromoter regions. The frequency of motif occurrence is shown, and the motifs are sorted by *P*-value. (*E*) Scatter bag plot of differential chromatin accessibility (*x*-axis) and footprinting (*y*-axis) depth around human transcription factor binding motifs in normal and Barrett's tissue. Significant outliers (*P* < 0.05) are represented in red. Motifs with enrichment in “open in Barrett's” and “closed in Barrett's” regions are labeled. (*F*) Plot of Log_2_(1 + FPKM) values of transcription factors with enriched motifs in differentially accessible regions from normal (blue) versus Barrett's analysis (“open in Barrett's”) in RNA-seq data from normal or Barrett's (black). Mean is represented by a black bar with standard deviation shown *above* and *below*. (****) *P* < 0.0001; (***) *P* < 0.001. (*G*) Heatmap of ATAC-seq signal from normal, Barrett's, and EAC tissue at differentially “open in Barrett's,” “closed in Barrett's,” and a control set of 500 random nonsignificant differential accessible regions. (*H*) UCSC Genome Browsers tracks of ATAC-seq signal at two example loci (*LGALS4* and *TFAP2B*) with differentially accessible regions boxed. (*I*) Expression of *LGALS4* and *TFAP2B* in normal, Barrett's, and EAC tissue (Maag et al. 2017). Mean is represented by a black bar with standard deviation shown *above* and *below*. (****) *P* < 0.0001; (***) *P* < 0.001.

EAC is thought to usually arise from a precancerous metaplastic state known as Barrett's esophagus (Desai et al. 2012). We therefore asked whether the regulatory network involving HNF4A and GATA6 could be detected in Barrett's esophageal tissue. First, we performed ATAC-seq on nondysplastic Barrett's tissue taken from four different patients. Data from these samples were highly consistent (Supplemental Fig. S8A). PCA analysis demonstrated that the Barrett's samples clustered together with the EAC samples rather than the samples from normal tissue (Fig. 4A). We therefore compared the open chromatin regions from Barrett's samples with those found in normal esophageal tissue (Supplemental Table S6). The majority (>98%) of changes in accessibility were found in nonpromoter regions, that is, intra- and intergenic regions with chromatin opening being the predominant (64%) change in Barrett's cells (Fig. 4B,C). “Gland development” was among the enriched GO terms in genes associated with opening chromatin regions, which is in keeping with the conversion of the stratified epithelium of the esophagus to a glandular epithelium in Barrett's cases (Supplemental Fig. S8C).

Next, to identify potential upstream regulatory proteins, we searched for enriched motifs in “open in Barrett's” nonpromoter regions and identified motifs for Forkhead-, GATA-, and HNF4-related transcription factors as the most enriched in the regions that are more accessible in Barrett's cells. Conversely, the TP53 binding motif is enriched in the regions that become less accessible in Barrett's cells (Fig. 4D; Supplemental Table S7). To ensure that these motifs are actually enriched in differentially accessible regions and not just accessible regions, we reanalyzed the “open in Barrett's” regions by using all accessible regions as background. Again, this analysis identified GATA, Forkhead, and HNF4 family motifs in regions becoming more accessible, and the TP53 motif in regions becoming more closed (Supplemental Fig. S8D). We also used BaGFoot (Baek et al. 2017) to identify motifs that showed evidence of changes of occupancy through altered “footprinting” in all the open chromatin regions in the Barrett's samples. Again, we identified motifs for GATA6 and Forkhead transcription factors, and in addition the motifs for HNF1A/B showed particularly strong increases in both footprinting depth across the motif and accessibility in the local surrounding area (Fig. 4E; Supplemental Fig. S9). Although the HNF4A motif was not identified using BaGFoot, centering accessible regions in Barrett's onto the HNF4A motif and plotting ATAC-seq signal across these regions shows a clear footprint in Barrett's cells compared to normal tissue (Supplemental Fig. S8E). It therefore appears that the same transcription factors that we identified in EAC are associated with the open chromatin regions in Barrett's esophageal cells. To identify the likely transcription factors binding to these sites, we examined the expression of several family members in Barrett's and normal esophageal tissue (Maag et al. 2017). GATA6, HNF4A/G, HNF1A/B, and FOXA1, FOXA2, and FOXA3 are all expressed to higher levels in Barrett's samples (Fig. 4F; Supplemental Fig. S8F). Given the prominent appearance of HNF4A and GATA6 binding motifs, we examined the expression of the genes in Barrett's samples that are directly regulated by HNF4A and GATA6 in OE19 cells. In keeping with a likely regulatory role for these transcription factors, these same sets of genes are also up-regulated in Barrett's tissue (Supplemental Fig. S8G).

Finally, as a direct comparison, we compared the open chromatin landscape of EAC cells with that found in Barrett's and normal esophageal tissue. Regions that are more accessible in Barrett's esophagus cells maintain this accessibility in EAC cells. Closed regions also maintain a similar state of reduced accessibility in EAC (Fig. 4G). Genome Browser tracks of the *LGALS4* (gained accessible region) and *TFAP2B* (lost accessible region) loci illustrate the “maintenance” of chromatin accessibility states in EAC compared to Barrett's esophagus (Fig. 4H). Expression of *LGALS4* and *TFAP2B* show higher or lower expression in Barrett's esophagus, respectively, and this level of expression is maintained in EAC, mirroring the loci accessibility patterns (Fig. 4I). To further assess the potential role of HNF4A and GATA6 initiating Barrett's esophagus, we examined ATAC-seq signal from normal, Barrett's, and EAC tissue at genomic regions bound by HNF4A alone, GATA6 alone, and regions cobound by GATA6 and HNF4A (Supplemental Fig. S8H). All regions show a large increase in accessibility from normal to EAC tissue. All regions also show a moderate increase in accessibility from normal to Barrett's, but regions with HNF4A (HNF4A alone and GATA6 and HNF4A cobound regions) show a much greater increase in accessibility during the normal-to-Barrett's transition and are also the most accessible in EAC compared to those with GATA6 binding alone. This suggests that HNF4A might initiate the opening of chromatin at these regions.

Together these data therefore indicate that the regulatory network involving HNF4A, GATA6, HNF1B, and FOXA transcription factors is already established in Barrett's metaplastic, is maintained in EAC cells, and HNF4A may initiate the development of Barrett's esophagus.

### HNF4A drives the formation of open chromatin

Our results are consistent with two possible models. Either one or more of the transcription factors in the regulatory network can bind to preconfigured open chromatin in Barrett's and EAC cells, or instead they might themselves directly trigger this opening. To test the latter possibility, we used lentivirus with doxycycline-inducible constructs to express either HNF4A or GATA6 in the “normal” esophageal Het1A cells and profiled the resulting open chromatin landscape using ATAC-seq after induction.

**Figure 5.**
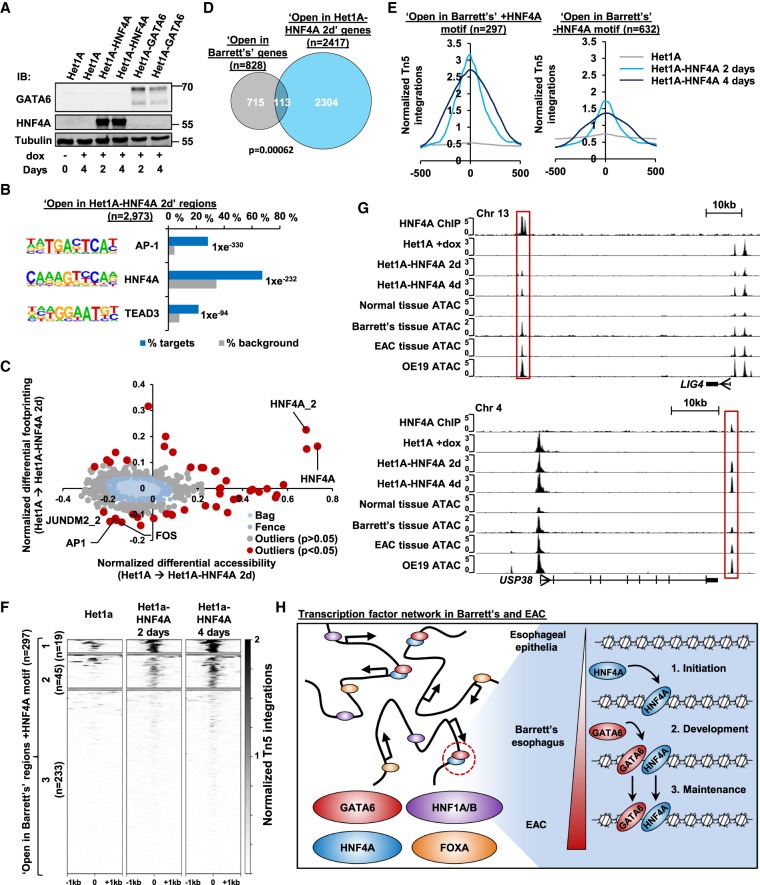
HNF4A demonstrates pioneer factor function. (*A*) Immunoblot analysis of HNF4A, GATA6, and tubulin alpha in Het1A, Het1A-HNF4A, and Het1A-GATA6 cells. The addition of doxycycline for 2 or 4 days is indicated (+). (*B*) The top three DNA motifs derived from de novo motif discovery of differentially accessible regions in Het1A-HNF4A cells after 2 d of induction and their associated transcription factors. The frequency of motif occurrence is shown, and the motifs are sorted by *P*-value. (*C*) Scatter bag plot of differential chromatin accessibility (*x*-axis) and footprinting (*y*-axis) depth around human transcription factor binding motifs in Het1A and Het1A-HNF4A cells. Significant outliers (*P* < 0.05) are represented in red. Motifs with enrichment in “open in Het1A-HNF4A” regions are labeled. (*D*) Overlap of genes annotated to “open in Barrett's” and “open in Het1A-HNF4A 2d” regions. *P*-value was calculated using the hypergeometric test. (*E*) Tag density plots of ATAC-seq signal from Het1A and Het1A-HNF4A cells (2 and 4 d doxycycline induction) around differentially open regions in Barrett's tissue with (*left*) or without (*right*) a HNF4A consensus binding motif. (*F*) Heatmap of ATAC-seq signal from Het1A and Het1A-HNF4A cells (2 and 4 d doxycycline induction) at “open in Barrett's” regions with a HNF4A motif. Regions were subjected to *k*-means hierarchical clustering (*k* = 3). (*G*) UCSC Genome Browser tracks of two example loci at *LIG4* and *USP38*, with regions boxed that show increased accessibility with HNF4A overexpression. (*H*) Model of the transcription factor network in Barrett's and EAC. HNF4A is able to promote chromatin opening in normal esophageal cells at a subset of Barrett's-specific regions. In Barrett's, a transcription factor network, including HNF4A and GATA6 are coexpressed, and these two transcription factors co-occupy a large number of genomic regions. This regulome persists in the context of the chromatin landscape of EAC cells.

Western blot and RT-qPCR analysis demonstrated induction of HNF4A and GATA6 protein and mRNA after 2 d of doxycycline treatment, and this was maintained at 4 d (Fig. 5A; Supplemental Fig. S10A). Ct values also indicated a similar expression of HNF4A and GATA6 upon induction (Supplemental Fig. S10A). ATAC-seq was then performed after 2 and 4 d of doxycycline treatment. All replicates had high levels of correlation (Supplemental Fig. S10B); thus, we merged the alignment files of the replicates of all samples from either the HNF4A expression or GATA6 expression time course and recalled peaks from each of these combined data sets. We then took the top 50,000 most significant regions to calculate differential accessibility between parental Het1A cells and Het1A-HNF4A and Het1A-GATA6 cells at 2 and 4 d of induction (Supplemental Table S8A,D). Differential accessibility analysis determined that HNF4A overexpression resulted in 2973 regions with increased accessibility and 976 regions with decreased accessibility after 2 d of induction. In contrast, after 2 d of induction of GATA6, only 87 regions became more accessible, and only 149 became less accessible. After 4 d of induction, the number of differential accessible regions remained similar for both transcription factors, so we focused on 2 d of induction to identify the immediate effects of transcription factor overexpression (Supplemental Fig. S10C; Supplemental Table S8A–F). Because HNF4A appears to be able to drive the formation of open chromatin much more robustly than GATA6, we decided to focus on the chromatin regions that are dynamically controlled by HNF4A. The genomic distribution of regions that become more accessible after HNF4A induction is similar to the HNF4A binding regions identified by HNF4A ChIP-seq (Supplemental Fig. S10D), and there was extensive overlap of accessible regions between 2 and 4 d of induction (Supplemental Fig. S10E). De novo transcription factor motif discovery in the chromatin regions that open up following HNF4A expression uncovered overrepresentation of binding motifs for AP-1, HNF4A, and TEAD (Fig. 5B; Supplemental Table S9). We next used BaGFoot to simultaneously look for differential accessibility and footprinting at these regions. The HNF4A motif showed both increased footprinting and increased accessibility (Fig. 5C; Supplemental Fig. S11). These data indicate that the differential accessible regions induced by HNF4A overexpression are indeed driven by HNF4A.

We next sought to investigate whether the regions of differential accessibility in Barrett's are associated with the HNF4A-induced events Het1A cells. First, we asked whether the genes associated with HNF4A-induced open chromatin regions showed any communalities in function to those found to increase in accessibility in EAC or Barrett's tissue samples. Similar biological processes are affected, and several GO terms were found to be in common such as “gland development” (Supplemental Fig. S10F). Next, we asked whether overlaps could be seen at the associated target gene level and uncovered a significant overlap in genes associated with the regions of increased accessibility (Fig. 5D). We then expanded this analysis by performing GO term analysis on this common set of genes (Supplemental Fig. S10G). Enriched GO terms included “digestive tract morphogenesis” in keeping with a role for HNF4A in inducing this phenotype.

Given these phenotypic overlaps, we next examined whether HNF4A promoted chromatin opening at regions that are found to be more accessible in Barrett's samples. We divided the “open in Barrett's” regions into those containing HNF4A motifs and then plotted the average normalized ATAC-seq signal from parental Het1A and Het1A-HNF4A at 2 and 4 d of induction. Regions with a motif showed a large increase of accessibility at 2 d and this was maintained at 4 d, whereas regions without a motif showed lower levels of increased accessibility (Fig. 5E). These findings are consistent with a direct role for HNF4A in promoting chromatin opening after engaging with its recognition element. To further delineate the induction of chromatin accessibility, we took all of the regions that contained an HNF4A binding motif and showed increased accessibility in Barrett's, centered them on the HNF4A motif, and plotted the tag densities of the ATAC-seq signals from the HNF4A induction profile in Het1A cells around the centers of these peaks. The profiles were then subjected to *k*-means clustering with three clusters, characterized by strong opening of chromatin (cluster 1), more moderate levels of chromatin opening (cluster 2), and little chromatin opening (cluster 3) (Fig. 5F; Supplemental Fig. S10H). Regions that show evidence of increased accessibility following HNF4A overexpression make up 64/298 (21%) of the open chromatin regions in Barrett's with a HNF4A motif. More generally, there is a significant overlap (*P* = 4.2 × 10^−6^) in the number of regions containing an HNF4A binding motif and showing increased accessibility in both Barrett's tissue and following HNF4A overexpression. Two example regions are associated with *LIG4* and *USP38*, which show an induction of chromatin opening with HNF4A, a region of accessibility in Barrett's, and EAC and direct evidence of HNF4A occupancy by ChIP-seq (Fig. 5G).

Collectively, these results indicate that HNF4A is capable of inducing chromatin opening in normal esophageal cells, and in this context, it is able to promote the opening of regions of chromatin that are seen to be differentially accessible in Barrett's and EAC. HNF4A therefore has the potential to promote a pivotal initiation event in Barrett's esophagus formation.

## Discussion

Cancer genome sequencing efforts have given us insights into the mutational spectrum and hence the potential molecular causes of EAC (Dulak et al. 2013; Weaver et al. 2014; Ross-Innes et al. 2015). These have contributed to a model in which EAC develops from a precancerous state known as Barrett's esophagus. However, in addition to genetic changes, epigenetic alterations to the chromatin landscape are also likely to play an important role in disease progression. Several recent studies have used ATAC-seq and open chromatin profiling to uncover the regulatory networks involved in cancer, such as in small cell lung cancer metastasis (Denny et al. 2016), ER-dependent breast cancer (Toska et al. 2017), and EMT (Pastushenko et al. 2018). Here, we used ATAC-seq to uncover the regulatory open chromatin landscape of EAC and nondysplastic Barrett's samples from patients and to demonstrate that they exhibit many similarities. This provides strong molecular support for Barrett's being a precursor state to EAC.

Previously, we identified AP-1 as an important regulatory transcription factor in EAC (Britton et al. 2017). Here, we took a more focused approach and investigated a subset of EAC samples, which enabled us to uncover a regulatory network comprised of HNF4A, GATA6, HNF1B, and FOXA1 transcription factors that exists in EAC but is already activated in Barrett's (Figs. 4D,E, 5H). These transcription factors have all been shown to play a role in intestinal development (Verzi et al. 2010; Beuling et al. 2012; Walker et al. 2014; Gosalia et al. 2015; San Roman et al. 2015; Yang et al. 2016), suggesting that Barrett's and EAC cells have reverted to a more primitive state in which these factors are operational. For example, HNF4A, GATA6, and FOXA3 are all broadly expressed in the stomach, liver, pancreas, and intestine during mouse embryogenesis but are absent from the esophagus (Sherwood et al. 2009). Similarly, we show that these factors are all expressed during early human intestinal development, but with the exception of early definitive endoderm, coexpression is not observed in any particular organ (Supplemental Fig. S3). An alternative hypothesis is that rare cells may exist that contain this regulatory network and may act, for example, as stem cells for replenishing the esophageal epithelium. In support of this, recent single-cell RNA-seq analysis of the esophageal epithelium revealed a population of esophageal submucosal gland cells that are transcriptionally similar to Barrett's esophagus and express HNF4A and GATA6 (Owen et al. 2018). By focusing on two of the highest expressed members of this network, HNF4A and GATA6, we showed that these two factors co-occupy many genomic loci and work together to control gene expression. However, it is likely that a much more complicated interacting network exists with multiple combinatorial interactions involving these two transcription factors, HNF1B and FOXA1. Indeed, in addition to the family members we have focused on, other related proteins such as HNF4G, GATA4, HNF1A, and FOXA2/3 are also expressed at higher levels in Barrett's and EAC. Therefore it is likely that there is functional redundancy built into the regulatory network, which may in part explain why the numbers of genes we find to be regulated by HNF4A and GATA6 is an order of magnitude lower than the number of direct targets. It is currently unclear how this network is initially established, but we demonstrated that HNF4A is able to penetrate and open regions of inaccessible chromatin, which may represent one of the initiating events for Barrett's development. However, it is important to emphasize that HNF4A is insufficient to drive the opening of all the newly accessible regions in Barrett's cells. Nevertheless, these results are consistent with the observation that HNF4A overexpression in mouse esophageal epithelial cells is sufficient to induce the expression of several Barrett's-specific markers (Colleypriest et al. 2017). This activity of HNF4A is akin to pioneering activity that has been demonstrated for several transcription factors (Zaret and Carroll 2011). However, GATA6 did not demonstrate widespread pioneering activity, and there is a distinct lack of GATA motifs in the HNF4A-induced accessible chromatin regions, despite our demonstration of widespread cobinding of HNF4 and GATA factors in Barrett's and EAC cells. This suggests that although HNF4A may be an initial driver, additional events must occur to facilitate subsequent GATA factor binding to expand the regulatory network. However, at this stage we cannot rule out a more widespread pioneering role for GATA factors at higher expression levels than we were able to achieve. One set of potential contributory factors would be the FOXA subfamily of Forkhead transcription factors that have been implicated in pioneering activity (Zaret and Carroll 2011) and, more generally, in promoting tumorigenesis (Lam et al. 2013). Previous studies have suggested a role for FOXA2 in promoting Barrett's metaplasia (Wang et al. 2014), and further studies are warranted to assess whether FOXA proteins act more widely in a pioneering capacity to drive this transition.

GATA4 and GATA6 were previously implicated in EAC by the numerous studies that have observed that their loci are frequently amplified in the transition from Barrett's esophagus to cancer (Lin et al. 2012; Stachler et al. 2015). However, we showed here that these transcription factors are already expressed in Barrett's and are associated with largely the same open chromatin regions in both Barrett's and EAC. This suggests that the supra-physiological levels of GATA4/6 arising from genomic amplifications may act on a different set of loci to drive EAC formation. Patient samples containing such amplifications are needed to test this hypothesis. GATA6 has also been shown to operate in gastric cancer (Sulahian et al. 2014; Chia et al. 2015), and recent genome sequencing data indicated that CIN variant gastric, GOJ, and EAC tumors are closely related at the molecular level (The Cancer Genome Atlas Research Network 2017). Our data therefore further support this conclusion. Indeed, the OE19 cells we used here to functionally validate a role for HNF4A and GATA6 were isolated from the GOJ, and these cells contain an open chromatin landscape that most closely resembles the data from patient-derived EAC samples.

HNF1A is known to respond to bile acids and was shown to up-regulate *MUC4* in EAC cells (Piessen et al. 2007). Furthermore, HNF4A has previously been implicated in the initial response of normal human esophageal mucosa to bile acids (Green et al. 2014). More recently, HNF4A was shown to be sufficient to induce a columnar-like phenotype in adult mouse esophageal epithelium (Colleypriest et al. 2017). These results suggest that HNF1A/B and HNF4A may play an initiating role in promoting the transition to Barrett's in human disease. Indeed, our results demonstrated that forced expression of HNF4A in normal esophageal epithelial cells is sufficient to trigger a chromatin opening that is observed in Barrett's and maintained in EAC. This provides further evidence to suggest that Barrett's might arise directly from the esophageal squamous epithelium. Alternatively, the same mechanisms might trigger the transition from other cell types that have been proposed as the cell of origin, including cells from the GOJ (Jiang et al. 2017) or migrating cells of gastric origin (Quante et al. 2012).

Although we uncovered a transcriptional regulatory network that clearly links EAC cases to underlying Barrett's, it is possible that cells may directly transition to EAC without transitioning through Barrett's. Indeed, two of the EAC cases that we studied do not possess the open chromatin landscape that is characteristic of Barrett's esophageal cells (Supplemental Fig. S1A; Britton et al. 2017). Further samples are needed to study alternative open chromatin landscapes and the underlying regulatory networks that might be established in EAC.

In summary, we have used open chromatin profiling to uncover the transcriptional regulatory networks that are operational in Barrett's esophagus and retained in EAC. This provides molecular insights into the stepwise progression toward EAC and implicates the reactivation of a set of transcription factors usually associated with primitive intestinal development from the HNF4, GATA, FOXA, and HNF1 subfamilies. This is therefore a potentially powerful approach to uncover regulatory pathways in cancer cells, stratify cancers, and identify biomarkers that can complement and extend the insights being provided through ongoing genomic sequencing efforts.

## Methods

### Ethics statement

Ethical approval for collection of Barrett's esophagus tissue samples from patients at Leigh Infirmary was granted by the ethics committee of Salford Royal NHS Foundation Trust (2010) (04/Q1410/57). Patient consent was obtained in written form and signed by the patient and doctor.

### Barrett's esophagus tissue collection

Barrett's esophagus samples of ∼2 mm were obtained from four consenting patients. All patients presented with at least C3M3 Barrett's, which is defined as “long” Barrett's and is of higher risk of progression to EAC (Fitzgerald et al. 2014). Intestinal metaplasia histology and no evidence of dysplasia was confirmed for all samples. Patient information can be found in Supplemental Table S10.

### Cell culture, siRNA transfections, and RNA isolation

OE19, Het1A, and HEK293T cells were grown, transfected with siRNA SMARTpool, RNA extracted, and RT-qPCR performed using established protocols (for details, see Supplemental Methods).

### HNF4A and GATA6 expressing stable Het1a cell line production

Lentiviral vectors containing HNF4A and GATA6 were constructed and then packaged and transfected into Het1A cells using established procedures (for details, see Supplemental Methods). Polyclonal cell lines were then selected with either G418 for Het1A-HNF4A cells (500 µg/mL) or Zeocin for Het1A-GATA6 cells (300 µg/mL) for 14 d. The expression of HNF4A and GATA6 was induced by treating Het1A-HNF4A or Het1A-GATA6 cells with 100 ng/mL doxycycline (Sigma-Aldrich). Parental Het1A cells were treated with 100 ng/mL doxycycline as a control for 4 d.

### Western blotting

Protein was isolated and western blotting was performed using anti-HNF4A (R&D Systems, PP-H1415-00), anti-GATA6 (Cell Signaling Technology, D61E4 XP), anti-α-Tubulin (Sigma-Aldrich, T9026) antibodies with IRDye secondary antibodies (Licor, 925-32212, 925-32213), and scanned with Odyssey IR scanner (Licor) (for details, see Supplemental Methods).

### RNA-seq analysis

RNA-seq libraries were prepared using a TruSeq-stranded mRNA sample prep kit and run on a HiSeq 4000 (Illumina) platform. Reading trimming, alignment to the genome, and differential expression analysis were performed using standard protocols (for details, see Supplemental Methods).

### ChIP, ChIP-seq, and ATAC-seq analysis

ChIP, ChIP-seq (Wiseman et al. 2015), and ATAC-seq (Garcia et al. 2016; Britton et al. 2017) were performed and analyzed essentially as described previously (for details, see Supplemental Methods). For ATAC-seq, differential accessible regions with a linear fold change > 5 and a *Q*-value < 0.05 were considered significant and were subject to further analyses.

### Bioinformatics analysis

To visualize ATAC-seq and ChIP-seq tag densities, tags were normalized by scaling libraries to 10 × 10^6^ tags (HOMER default) and counted using HOMER v4.7 annotatePeaks.pl with –hist parameter (Heinz et al. 2010) and plotted in Microsoft Excel. Chromatin accessibility and gene expression heatmaps at individual loci/genes were drawn using Morpheus (https://software.broadinstitute.org/morpheus/), and hierarchical clustering was performed with this software using 1-Pearson's correlation unless otherwise stated. ChIP-seq heatmaps and correlation plots were drawn using deepTools2 v2.5.2 (Ramírez et al. 2016). De novo motif analysis was performed using HOMER v4.7 findMotifsGenome.pl with –cpg and –mask parameters, and motif counting within regions was also performed using HOMER v4.7 annotatePeaks.pl with –m parameter (Heinz et al. 2010). ATAC-seq and ChIP-seq gene annotation to hg19 were performed using HOMER annotatePeaks.pl, implementing the closest gene model. Gene Ontology (GO) analysis was performed using Metascape (http://metascape.org) (Tripathi et al. 2015). Principal component analysis scores were calculated using prcomp (x, scale = T, center = T) in R (R Core Team 2018) from log_2_ transformed ATAC counts in the top 50,000 accessible regions ranked by *Q*-value and were plotted using Microsoft Excel. Correlation plots were generated using the R package Corrplot (https://github.com/taiyun/corrplot). Footprinting analysis on a subset of ATAC peaks was performed using the Wellington footprinting algorithm in the pyDNase package v0.2.4 (Piper et al. 2013), and genome-wide simultaneous differential accessibility and footprinting analysis was performed using BaGFootR v0.9.7 (Baek et al. 2017). Venn diagrams were visualized using the R package eulerr (http://eulerr.co/). For sources of published RNA-seq and ATAC-seq, see Supplemental Methods.

### Statistical analysis

To determine statistical significance between two groups, a Student's unpaired two-tailed *t*-test was performed using GraphPad Prism v7. To assess the significance of motif co-occurrence distributions, a χ^2^ test was performed in GraphPad Prism v7. To assess the significance of gene/region overlaps, a hypergeometric distribution test was performed using the phyper function in R. *P*-values < 0.05 were considered significant.

## Data access

Data generated in this study have been submitted to ArrayExpress (https://www.ebi.ac.uk/arrayexpress/) under accession numbers E-MTAB-6751 and E-MTAB-6931 (ATAC-seq data), E-MTAB-6858 (ChIP-seq data), and E-MTAB-6756 (RNA-seq data).

## Supplementary Material

Supplemental Material
