# Supplementary material for: Identification of a primitive intestinal transcription factor network shared between esophageal adenocarcinoma and its precancerous precursor state
Source: Genome Res. 2019 May;29(5):723–36. doi: 10.1101/gr.243345.118 (PMC6499311; doi:10.1101/gr.243345.118)
Supplement: Supplemental Material [file supp_29_5_723__index.html]

Identification of a primitive intestinal transcription factor network shared between esophageal adenocarcinoma and its precancerous precursor state — Supplemental Material 

# Identification of a primitive intestinal transcription factor network shared between esophageal adenocarcinoma and its precancerous precursor state

## Supplemental Material

- Supplemental\_Table\_S1.xlsx
- Supplemental\_Table\_S2.xlsx
- Supplemental\_Table\_S3.xlsx
- Supplemental\_Table\_S4.xlsx
- Supplemental\_Table\_S5.xlsx
- Supplemental\_Table\_S6.xlsx
- Supplemental\_Table\_S8.xlsx
- Supplemental\_Materials\_Methods.docx
- Supplemental\_Figures\_Tables.pdf
