# Supplementary material for: Identification of a primitive intestinal transcription factor network shared between esophageal adenocarcinoma and its precancerous precursor state
Source: Genome Res. 2019 May;29(5):723–36. doi: 10.1101/gr.243345.118 (PMC6499311; doi:10.1101/gr.243345.118)
Supplement: Supplemental Material [file supp_gr.243345.118_Supplemental_Materials_and_Methods_.docx]

**Supplementary Methods**

**Cell culture:** All cells were cultured in an incubator maintained at 37°C and 5% CO_2_. OE19 cells were cultured in RPMI 1640 media (Gibco) supplemented with 10% FBS (Gibco) and 1% penicillin-streptomycin (Gibco). Het1A and HEK293T cells were cultured in DMEM media (Gibco) with 10% FBS and 1% penicillin-streptomycin. Human tissue was processed as described previously (Britton et al., 2017). The expression of HNF4A and GATA6 was induced by treating Het1A-HNF4A or Het1A-GATA6 cells with 100 ng/mL doxycycline (Sigma). Parental Het1A cells were treated with 100ng/mL doxycycline as a control for 4 days.

**RNA isolation and RT-qPCR:** RNA was isolated from 2 x 10^5^ cells using a RNeasy RNA extraction kit (Qiagen, 74136) as per the manufacturer’s instructions. RT-qPCR was carried out using QuantiTect SYBR® Green RT-PCR Kit (Qiagen) using the primer pairs detailed in Table S11. Relative gene expression was calculated using the ΔΔCT method relative to levels of *RPLP0* mRNA.

**siRNA transfection:** SMART-pool siRNAs against *HNF4A* (Dharmacon, L-003406-00-0005), *GATA6* (Dharmacon*,* L-008351-00-0005), and control non-targting siRNA (siNT; Dharmacon, D-001810-10-0020) were transfected using Lipofectamine® RNAiMAX Transfection Reagent (Thermo Fisher Scientific, 13778150) as per the manufacturer’s instructions. Briefly, 2 x 10^5^ cells were transfected with 100 pmol siRNA and RNA was harvested 72 hours later.

**Lentivirus vectors, transduction and stable cell line production:** A lentiviral vector containing HNF4A was generated by amplifying HNF4A from pcDNA3.1-HNF4A8 (a gift from Frances Sladek; Vuong et al., 2015) with primers containing restriction enzyme sites for BamHI and EcoRI (5’-GATCGGATCCCCCACCATGGTCAGCGTG-3’, ADS6352; 5’-GATCGAATTCCGCTAGATAACTTCCTGCTT-3’, ADS6353). The PCR fragment was inserted into pENTR1A and transferred to pInducer20 by Gateway cloning to create pAS4393. A lentiviral vector containing GATA6 (pSLIKneo 3XFLAG-wtGATA6-3XAU1_RNAi resistant) was a gift from Kevin Janes (Addgene, 72616; Chitforoushzadeh et al., 2016). To move the positioning of the *BleoR* gene so it was included in the integration cassette, the *BleoR* gene was amplified from this plasmid using primers containing restriction enzyme sites for XhoI and PacI (5’- GATCCTCGAGGTGTGTCAGTTAGGGTGTGG-3’, ADS6354; 5’- GATCTTAATTAATCGAAATCTCGTAGCACGTG-3’, ADS6355) and inserted back into pSLIKneo 3XFLAG-wtGATA6-3XAU1_RNAi resistant using XhoI and PacI sites to create pAS4395. Lentiviruses were produced as previously described (Nowicki-Osuch et al., 2017). Briefly, HEK293T cells were transfected with pMD2.G (Addgene, 12259), psPAX2 (Addgene, 12260) and the target plasmid using PolyFect (Qiagen, 301107). Viral particles were precipitated from media using PEG-it (System Biosciences, LV810A-1) and Het1A cells were transduced by viral particles using Polybrene (EMD Millipore, TR-1003) as the transduction reagent. Het1A cells containing viral particles were selected with either G418 for Het1A-HNF4A cells (500 µg/mL) or Zeocin for Het1A-GATA6 cells (300 µg/mL) for 14 days to ensure that all non-transduced cells were dead. Selected cells were maintained in DMEM containing either 250 µg/mL G418 (Thermo Fisher Scientific, 10131027) or 100 µg/mL Zeocin™ (Thermo Fisher Scientific, R25001).

**Protein isolation and analysis:** Protein was isolated from 2 x 10^5^ cells by lysing cells in RIPA buffer on ice and sonicating for 5 minutes 30s on/off (Diagenode bioruptor). Protein was quantified using Pierce™ BCA Protein Assay Kit (ThermoFisher, 23227). Equal amounts were run on 10% poly-acrylamide gel electrophoresis gels and transferred to Amersham Protran nitrocellulose membrane (GE life sciences, 1060002). Membranes were blocked in Odyssey® Blocking Buffer (PBS) (Licor, 927-4000), probed with anti-HNF4A (R&D Systems, PP-H1415-00), anti-GATA6 (CST, D61E4 XP), anti-Tubulin (Sigma, T9026) antibodies with IRDye® secondary antibodies (Licor, 925-32212, 925-32213) and scanned with Odyssey® IR scanner (Licor).

**Aligning reads to genome:** Throughout this paper we used the reference hg19 genome (see below for details of mapping various datasets). When this project started in 2013, our initial ATAC-seq samples were mapped to hg19. Rather than remapping everything, we built on this original data and we do not think that re-aligning to GRCh38 will significantly affect our conclusions for several reasons; Generally, the changes between GRCh38 and hg19 are less than 1%; The majority of the improvements are to repetitive regions which are filtered out in our analyses; Most of our conclusions are based on the locations of regulatory elements and associations with nearby target genes, and their relative positions are unlikely to be changed between hg19 and GRCh38.

**RNA-seq analysis:** RNA-seq libraries were prepared using a TruSeq stranded mRNA sample prep kit and run on a HiSeq 4000 (Illumina) platform. Illumina adapters were trimmed using Trimmomatic v0.32 (Bolger et al., 2014). Trimmed reads were aligned to the RefSeq transcript annotation of human genome 19 (hg19) using STAR aligner v2.3.0e (Dobin et al., 2013). Reads mapping to Chromosomes 1-22, X and Y were retained. Gene expression levels (FPKMs) were estimated using CuffNorm (default parameters; geometric normalisation) from the Cufflinks package v2.2.1 (Trapnell et al., 2012). Differential expression analysis was carried out using CuffDiff (default parameters; geometric normalisation) from the Cufflinks package v2.2.1.

**ChIP and ChIP-seq analysis:** ChIP and ChIP-seq was carried out as described previously (Wiseman et al., 2015). For ChIP-qPCR, 5 x 10^6^ cells, 20 μl appropriate Dynabeads™ (Protein A beads for rabbit IgG and Protein G beads for mouse IgG; Thermo Fisher Scientific, 10004D/10002D) and either 1 μg HNF4A antibody (R&D Systems, PP-H1415-00), GATA6 antibody (CST, D61E4 XP) was used. PCR was carried out using the primer pairs detailed in Table S10. For ChIP-seq, 1 x 10^7^ cells, 2.5 µg antibody and 50 μl Dynabeads™ were used. Either normal rabbit IgG (Millipore, 12-370) or normal mouse IgG (Millipore, 12-371) antibody was used in parallel as control experiments. DNA libraries were prepared using TruSeq ChIP sample prep kit (Illumina) and sequenced on a HiSeq 4000 (Illumina) platform. Sequencing reads were aligned to NCBI Build hg19 using Bowtie 2 v2.2.3 (Langmead et al., 2009). Only reads with a mapping quality >q30 were retained. Peak calling was performed on individual replicates using MACS2 v2.1.1 (Zhang et al., 2008) using default parameters with additional –SPMR parameter. The overlap of peaks between two independent biological datasets was calculated using BEDTools v2.26.0 (Quinlan and Hall, 2010) using BEDTools intersect with default settings and using replicate one as a reference. Peaks present in both datasets were taken forward for further analysis.

**ATAC-seq and analysis:** ATAC-seq was performed as described previously (Britton et al., 2017). Differential accessibility was determined by merging alignment files of all datasets from both conditions and peaks recalled using MACS2 v2.1.1 (Zhang et al., 2008). The top 50,000 regions ranked by q-value were taken forward, a region +/- 250bp was taken around the peak summit and regions were separated by promoter (-2.5kb,+0.5kb of TSS) and non-promoter regions. Promoter and non-promoter regions were tested for differential accessibility using CuffDiff (default parameters; geometric normalisation) from the Cufflinks package v2.1.1 (Trapnell et al., 2012). Differential accessible regions with a linear fold change of >5 and a q-value of <0.05 were considered significant and were subject to further analyses.

**Published datasets used:** Human oesophagus, Barrett’s, high-grade dysplastic Barrett’s and OAC RNA-seq was sourced from ArrayExpress (E-MTAB-4054)(Maag et al., 2017). Additional OAC RNA-seq data was obtained directly from TCGA (<https://tcga-data.nci.nih.gov/docs/publications/esca_2017/>; Cancer Genome Atlas Research Network, 2017). ATAC-seq data from human oesophageal tissue (normal and OAC), and oesophageal cell lines was obtained from E-MTAB-5169 (Britton et al., 2017) and E-MTAB-4209 (Garcia et al., 2016).

**Statistical analysis:** To determine statistical significance between two groups, a Student’s unpaired two-tail T-test was carried out using GraphPad Prism v7. To assess the significance of motif co-occurance distributions, a Chi-squared test was carried out in GraphPad Prism v7. To assess the significance of gene/region overlaps, a hypergeometric distribution test was carried out using the phyper function in R. P-values <0.05 were considered as significant.

**Supplementary References**

Bolger AM, Lohse M, Usadel B. 2014. Trimmomatic: A flexible trimmer for Illumina sequence data. *Bioinformatics* **3:** 2114–2120.

Chitforoushzadeh Z, Ye Z, Sheng Z, LaRue S, Fry RC, Lauffenburger DA, and Janes KA. 2016. TNF-insulin crosstalk at the transcription factor GATA6 is revealed by a model that links signaling and transcriptomic data tensors. *Sci Signal*. **9**: ra59.

Dobin A, Davis CA, Schlesinger F, Drenkow J, Zaleski C, Jha S, Batut P, Chaisson M, Gingeras TR. 2013. STAR: ultrafast universal RNA-seq aligner. *Bioinformatics*. **29**: 15-21.

Garcia E, Hayden A, Birts C, Britton E, Cowie A, Pickard K, Mellone M, Choh C, Derouet M, Duriez P, Noble F, White MJ, Primrose JN, Strefford JC, Rose-Zerilli M, Thomas GJ, Ang Y, Sharrocks AD, Fitzgerald RC, Underwood TJ; OCCAMS consortium. (2016) Authentication and characterisation of a new oesophageal adenocarcinoma cell line: MFD-1. *Sci Rep*. **6**: 32417.

Langmead B, Trapnell C, Pop M, Salzberg S. 2009. Ultrafast and memory-efficient alignment of short DNA sequences to the human genome. *Genome Biol*. **10**: R25.

Nowicki-Osuch K, Li Y, Challinor M, Gerrard DT, Hanley NA, Sharrocks AD. 2017. EINCR1 is an EGF inducible lincRNA overexpressed in lung adenocarcinomas. *PLoS ONE* **12**: e0181902.

Quinlan AR, Hall IM. 2010. BEDTools: A flexible suite of utilities for comparing genomic features. *Bioinformatics* ***26*:** 841–842.

Trapnell C, Roberts A, Goff L, Pertea G, Kim D, Kelley DR, Pimentel H, Salzberg SL, Rinn JL, Pachter L. 2012. Differential gene and transcript expression analysis of RNA-seq experiments with TopHat and Cufflinks. *Nat Protoc*. ***7*:** 562–578.

Vuong LM, Chellappa K, Dhahbi JM, Deans JR, Fang B, Bolotin E, Titova NV, Hoverter NP, Spindler SR, Waterman ML, et al. (2015) Differential Effects of Hepatocyte Nuclear Factor 4α Isoforms on Tumor Growth and T-Cell Factor 4/AP-1 Interactions in Human Colorectal Cancer Cells. *Mol Cell Biol*. **35**:3471-90.

Zhang Y, Liu T, Meyer CA, Eeckhoute J, Johnson DS, Bernstein BE, Nussbaum C, Myers RM, Brown M, Li W, et al. 2008. Model-based analysis of ChIP-Seq (MACS). *Genome Biol.* **9**: R137.
