# Supplementary material for: Identification of a primitive intestinal transcription factor network shared between esophageal adenocarcinoma and its precancerous precursor state
Source: Genome Res. 2019 May;29(5):723–36. doi: 10.1101/gr.243345.118 (PMC6499311; doi:10.1101/gr.243345.118)
Supplement: Supplemental Material [file supp_gr.243345.118_Supplemental_Figures_Tables.pdf]

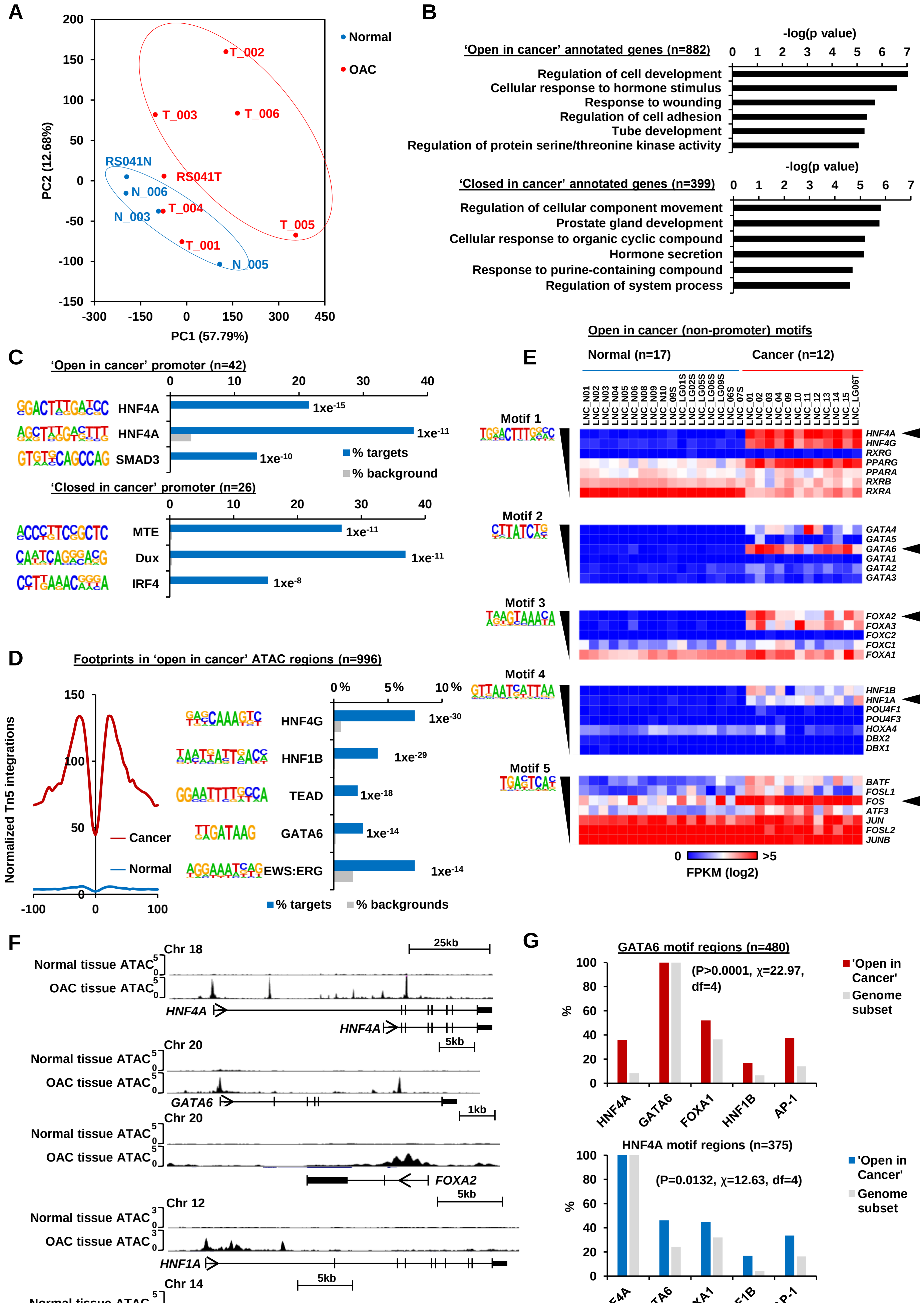

A
Normal
OAC
PC2 (12.68%)
PC1 (57.79%)
T_002
T_003
T_006
RS041N
RS041T
N_006
T_004
N_003
T_005
T_001
N_005
B
-log(p value)
'Open in cancer' annotated genes (n=882)
Regulation of cell development
Cellular response to hormone stimulus
Response to wounding
Regulation of cell adhesion
Tube development
Regulation of protein serine/threonine kinase activity
'Closed in cancer' annotated genes (n=399)
Regulation of cellular component movement
Prostate gland development
Cellular response to organic cyclic compound
Hormone secretion
Response to purine-containing compound
Regulation of system process
C
'Open in cancer' promoter (n=42)
HNF4A 1xe-15
HNF4A 1xe-11
SMAD3 1xe-10
% targets
% background
'Closed in cancer' promoter (n=26)
MTE 1xe-11
Dux 1xe-11
IRF4 1xe-8
D
Footprints in 'open in cancer' ATAC regions (n=996)
Normalized Tn5 integrations
Cancer
Normal
HNF4G 1xe-30
HNF1B 1xe-29
TEAD 1xe-18
GATA6 1xe-14
EWS:ERG 1xe-14
% targets
% backgrounds
E
Open in cancer (non-promoter) motifs
Normal (n=17)
Cancer (n=12)
Motif 1
HNF4A HNF4G RXRG PPARG PPARA RXRB RXRA
Motif 2
GATA4 GATA5 GATA6 GATA1 GATA2 GATA3
Motif 3
FOXA2 FOXA3 FOXC2 FOXC1 FOXA1
Motif 4
HNF1B HNF1A POU4F1 POU4F3 HOXA4 DBX2 DBX1
Motif 5
BATF FOSL1 FOS ATF3 JUN FOSL2 JUNB
0 >5
FPKM (log2)
F
Chr 18
25kb
Normal tissue ATAC
OAC tissue ATAC
HNF4A
HNF4A
Chr 20
5kb
GATA6
Chr 20
1kb
FOXA2
Chr 12
5kb
HNF1A
Chr 14
5kb
FOS
G
GATA6 motif regions (n=480)
(P>0.0001, χ=22.97, df=4)
'Open in Cancer'
Genome subset
HNF4A motif regions (n=375)
(P=0.0132, χ=12.63, df=4)
HNF4A
GATA6
FOXA1
HNF1B
AP-1

Fig. S1

Figure S1. Open chromatin profiling of OAC tissue samples. (A) PCA plot of normalised ATAC-seq signal from the top 50,000 regions of 4 normal tissue (blue) and 7 tumour tissue (red). (B) Top three motifs and their matched transcription factor from *de novo* motif discovery of 'open in cancer' and 'closed in cancer' promoter regions. The frequency of motif occurrence is shown and motifs are sorted by p-value. (C) GO term analysis of genes associated with 'open in cancer' and 'closed in cancer' differentially accessible regions. (D) Normalised Tn5 integrations from normal (blue) and cancer (red) tissue around called footprints (using pyDNase; Piper et al., 2013) within regions that are differentially accessible and 'open in cancer' (left). The top five *de novo* motifs identified within these footprint containing open regions, is shown as frequency of occurrence and sorted by p-value (right). (E) Heatmaps of log_2(1+FPKM) values of transcription factors with enriched DNA binding motifs (shown on the left) in 'open in cancer' regions in RNAseq data from normal or OAC tissue (Maag et al., 2017). Hierarchical clustering of samples was performed using 1-Pearson's correlation and rows sorted by average fold change in cancer (depicted by triangle). Arrows indicate the highest expressed factor in OAC. (F) UCSC genome browser tracks of *HNF4A*, *GATA6*, *FOXA2*, *HNF1A* and *FOS* showing ATAC-seq signal from normal and OAC tissue. (G) Bar chart of the percentage of 'Open in Cancer' regions that contain either a GATA6 (top) or HNF4A (bottom) motif and additionally contain a motif for either HNF4A, GATA6, FOXA1, HNF1B or AP-1. A random selection of HNF4A and GATA6 motifs in the genome were also assessed for presence of HNF4A, GATA6, FOXA1, HNF1B or AP-1 motifs. P-values, χ value and df are shown for each chart.

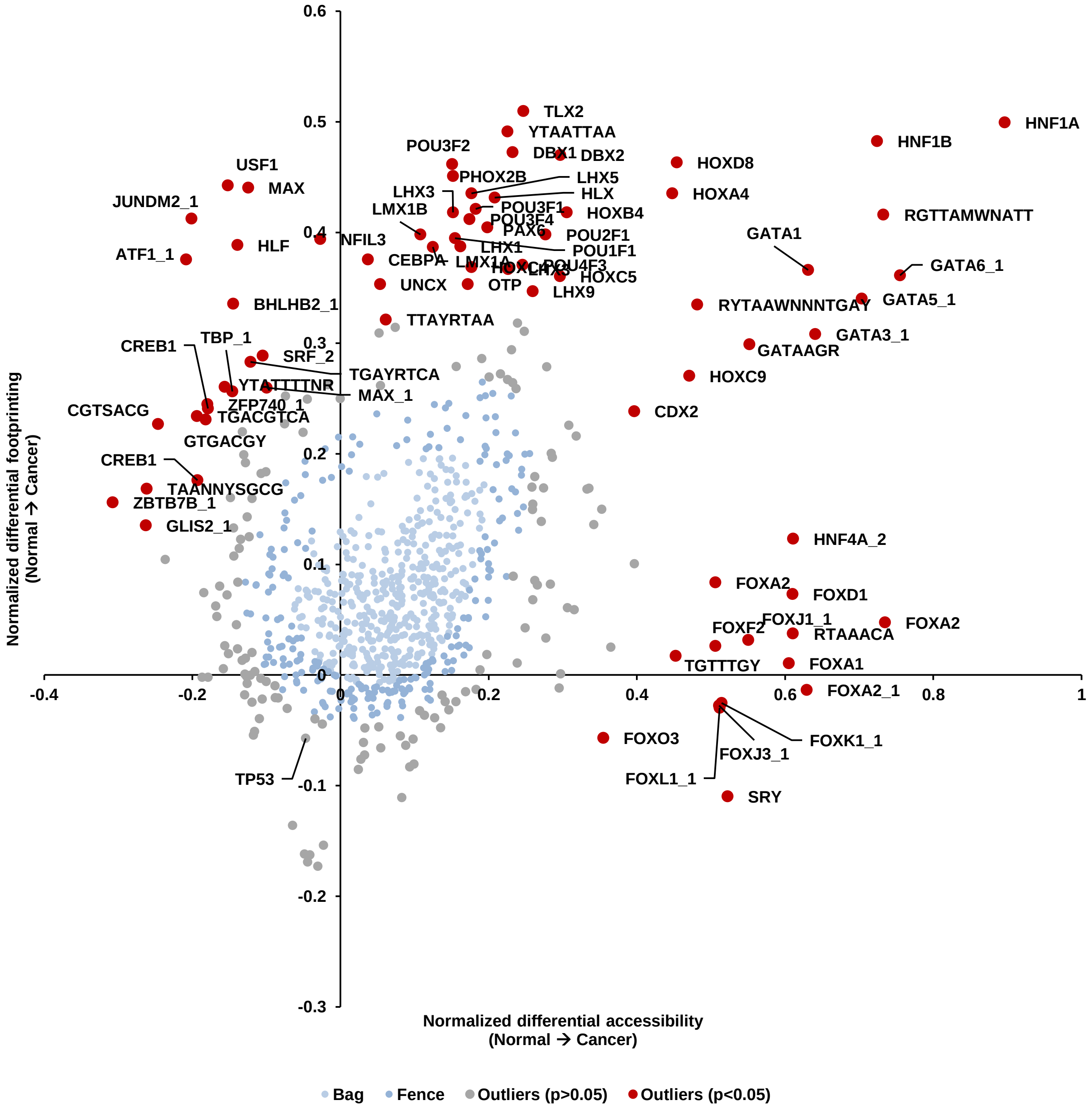

Figure S2. BaGFoot analysis of open chromatin regions in OAC tissue. Scatter bag plot of differential chromatin accessibility (x-axis) and footprinting (y-axis) depth around human transcription factor binding motifs in normal and cancer tissue. Significant outliers ($p<0.05$) are represented in red. This is identical to Fig. 1G but with full labelling of the motifs included.

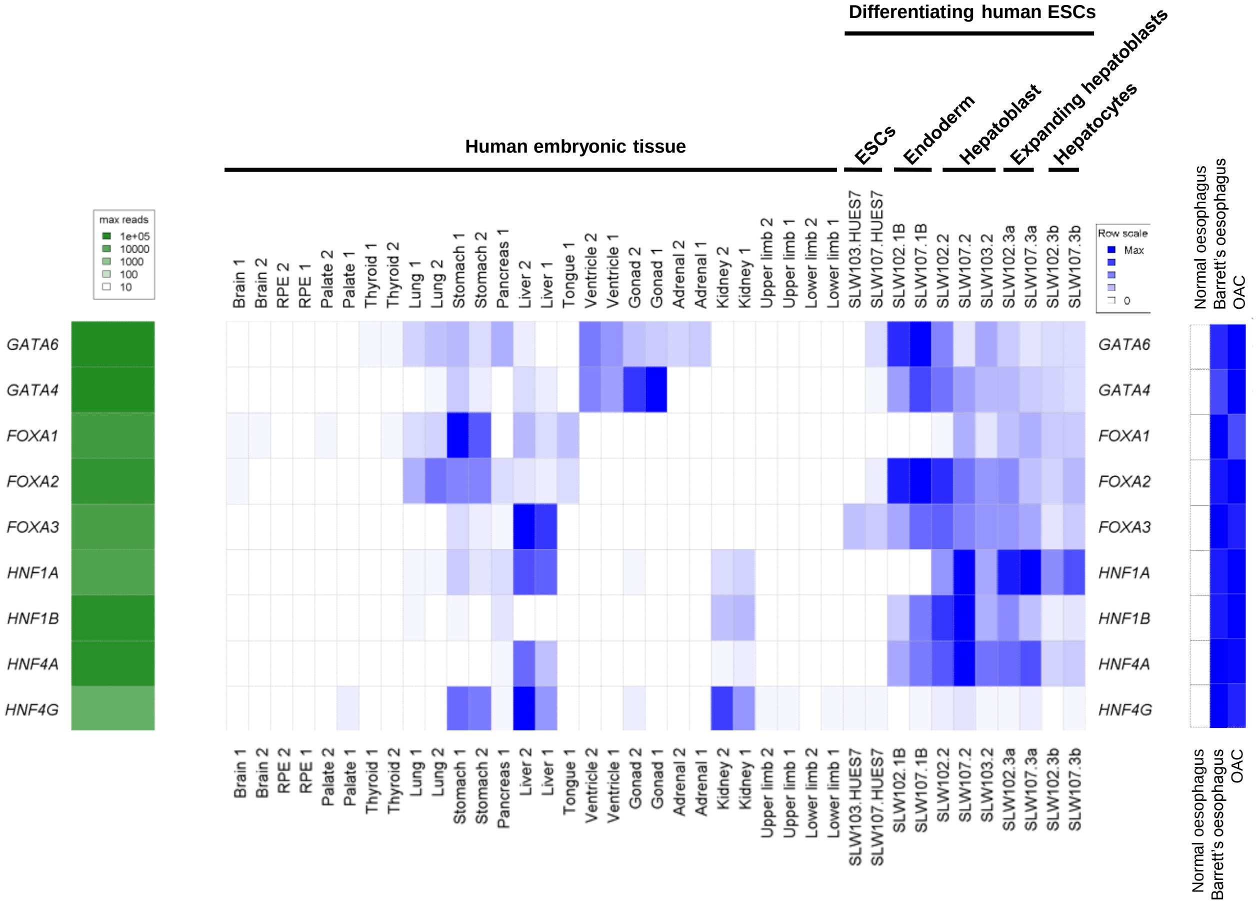

Figure S3. Expression of OAC-specific transcription factors during embryonic human intestinal development. Heatmap showing the expression of the indicated transcription factors across the indicated embryonic tissues and differentiated cell types derived from HUES7 embryonic stem cells (left) and expressed in normal, Barrett's and OAC tissue (right). Data are row Z normalized. The maximal number of reads found in one of the tissue/cell samples is shown on the left.

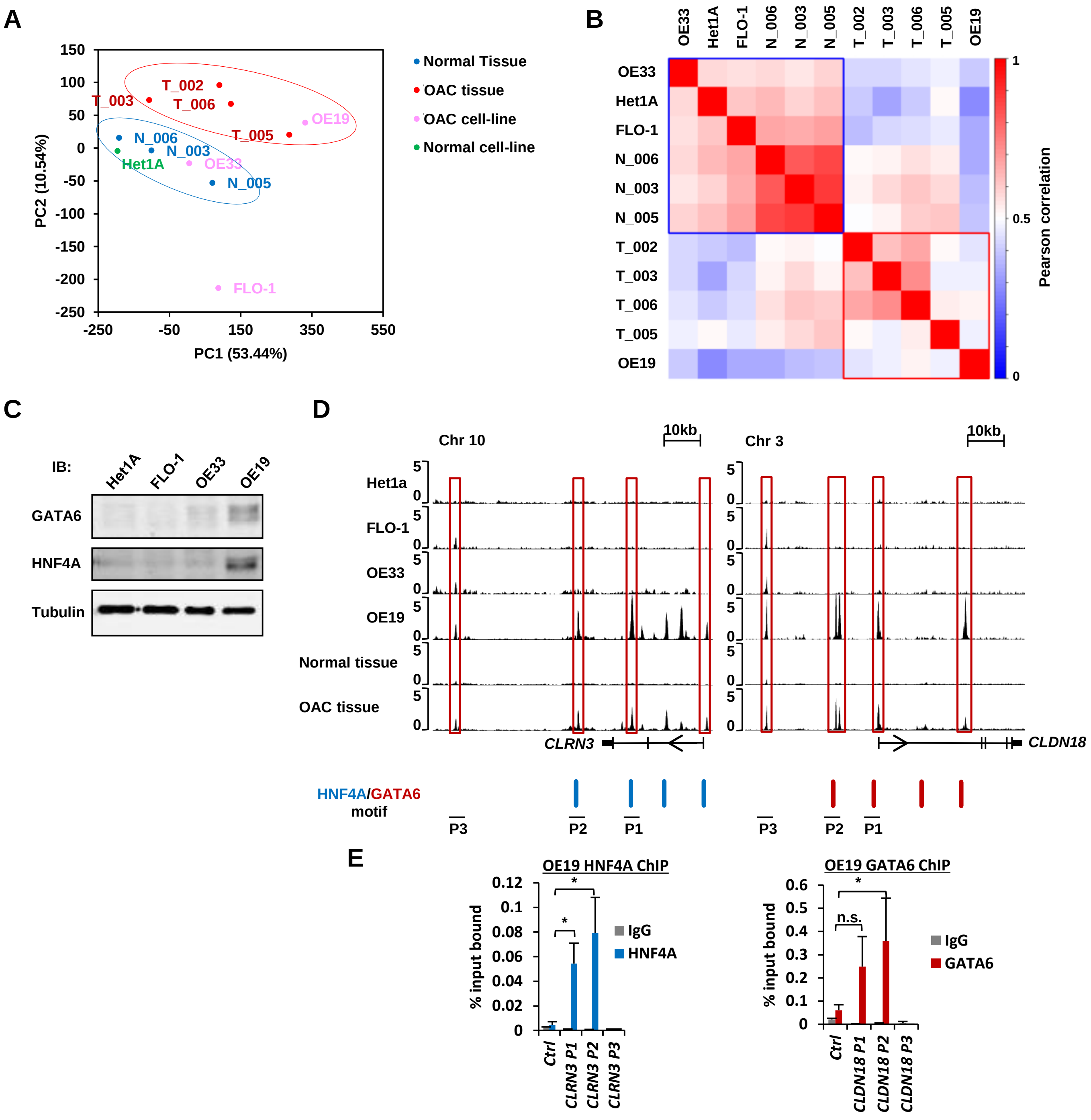

Figure S4. The chromatin landscape of OE19 cells represents OAC OAC tissue. (A) PCA plot of ATAC-seq signal from the top 50,000 ATAC-seq regions of data from normal tissue (blue), OAC tissue (red), three OAC cell lines OE33, OE19 and FLO-1 (pink) and a normal oesophageal cell line Het1A (green). (B) Pearson correlation plot of the top 50,000 ATAC-seq regions from normal (N_), OAC (T_) and the indicated cell line samples. Samples were clustered hierarchically and the two main clusters are highlighted as a 'normal' cluster (blue) and a 'cancer' cluster (red). (C) Immunoblot for GATA6, HNF4A and Tubulin expression in Het1A, FLO-1, OE33 and OE19 cell lysates. (D) UCSC genome browser tracks showing ATAC-seq signals of two loci surrounding the *CLRN3* and *CLDN18* genes. Common ATAC-seq peaks found in OAC tissue and OE19 cells are highlighted in red. The presence of a HNF4A and GATA6 binding motifs is indicated in blue and red below the peaks. (E) ChIP-qPCR analysis of the indicated ATAC-seq peak regions for HNF4A (left) or GATA6 (right) occupancy in OE19 cells. The identities of the regions tested are shown at the bottom of (D). * represents $p < 0.05$ (n=3).

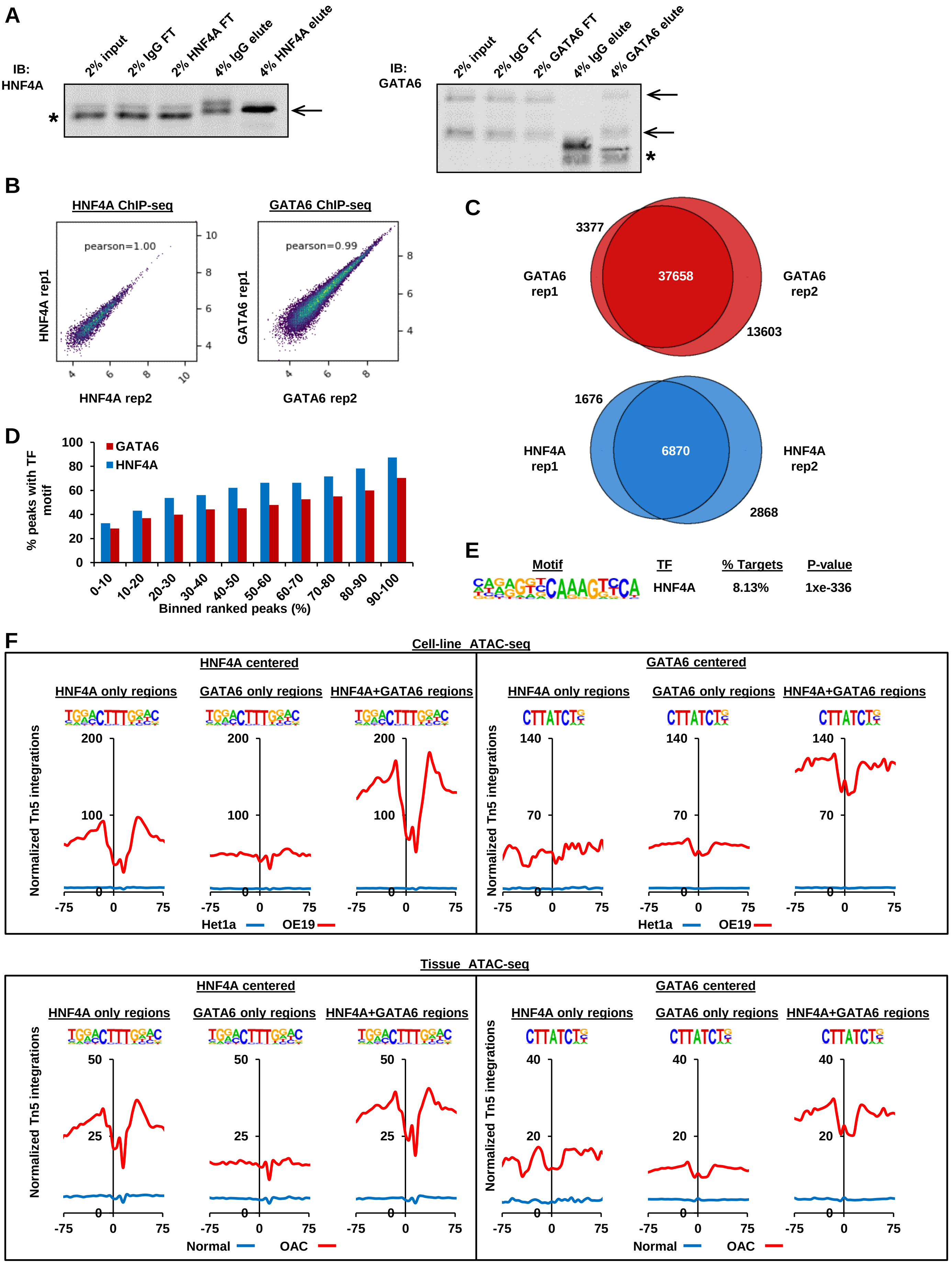
A
IB: HNF4A
2% input
2% IgG FT
2% HNF4A FT
4% IgG elute
4% HNF4A elute
*
IB: GATA6
2% input
2% IgG FT
2% GATA6 FT
4% IgG elute
4% GATA6 elute
*
B
HNF4A ChIP-seq
pearson=1.00
HNF4A rep1
HNF4A rep2
GATA6 ChIP-seq
pearson=0.99
GATA6 rep1
GATA6 rep2
C
3377
GATA6 rep1
37658
GATA6 rep2
13603
1676
HNF4A rep1
6870
HNF4A rep2
2868
D
GATA6
HNF4A
% peaks with TF motif
Binned ranked peaks (%)
0-10
10-20
20-30
30-40
40-50
50-60
60-70
70-80
80-90
90-100
E
Motif
TF
% Targets
P-value
HNF4A
8.13%
1xe-336
F
Cell-line ATAC-seq
HNF4A centered
GATA6 centered
HNF4A only regions
GATA6 only regions
HNF4A+GATA6 regions
Normalized Tn5 integrations
Het1a
OE19
Tissue ATAC-seq
Normal
OAC

FIG.S5

Figure S5. HNF4 and GATA6 ChIP-seq analysis. (A) Immunoblots of HNF4A (left) and GATA6 (right) following ChIP in different fractions; IgG FT (flow through from non-specific IgG precipitation), HNF4A/GATA6 FT (flowthrough from HNF4A/GATA6 precipitation), IgG elute (material eluted following IgG precipitation) HNF4A/GATA6 elute (material eluted following HNF4A/GATA6 precipitation). Arrows indicating immunoprecipitated protein (HNF4A/GATA6) and asterisks are non-specific cross-reacting bands (left) or the IgG heavy chain (right). (B) Scatter plot of ChIPseq signal between experimental replicates for HNF4A and GATA6 with Pearson correlation indicated. (C) Overlap of peaks called by each replicate for HNF4A and GATA6 ChIP-seq. The numbers of peaks in each sector are given. (D) Percentage of HNF4A and GATA6 ChIP-seq peaks with a HNF4A or GATA6 motif respectively. Peaks were sorted on enrichment and separated into bins, each containing 10% of the peaks. The presence of a HNF4A and GATA6 motif was assessed in each bin, with values ranging from 87%-32% for HNF4A and 70%-28% for GATA6. (E) Presence of HNF4A motif in GATA6 ChIP-seq regions. (F) Footprinting of ATAC-seq data from OE19 and Het1A cell-lines (top) or normal and OAC tissue (bottom) on 150 bp regions surrounding the HNF4A (left) or GATA6 (right) transcription factor motifs found in HNF4A-only, GATA6-only and HNF4A+GATA6 co-occupied ChIP-seq regions. Normalised Tn5 integrations are plotted across each of the regions relative to the centre of the binding motif. Data from cancer cells/tissue are shown in red and normal cells/tissue in blue.

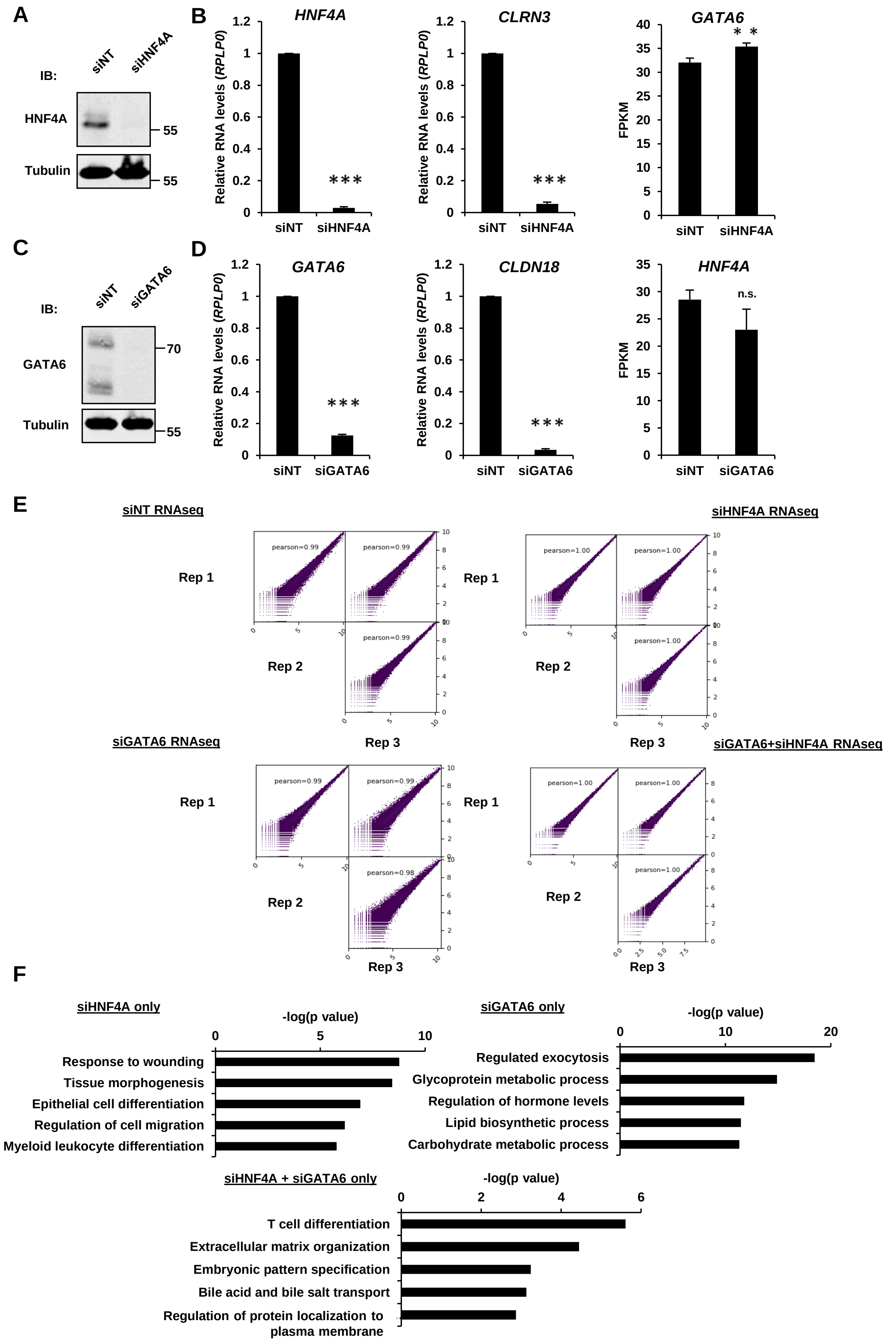
A
IB:
siNT
siHNF4A
HNF4A
55
Tubulin
55
B
HNF4A
CLRN3
GATA6
Relative RNA levels (RPLP0)
FPKM
siNT
siHNF4A
***
**
C
IB:
siNT
siGATA6
GATA6
70
Tubulin
55
D
GATA6
CLDN18
HNF4A
Relative RNA levels (RPLP0)
FPKM
siNT
siGATA6
***
n.s.
E
siNT RNAseq
siHNF4A RNAseq
siGATA6 RNAseq
siGATA6+siHNF4A RNAseq
Rep 1
Rep 2
Rep 3
pearson=0.99
pearson=1.00
pearson=0.98
F
siHNF4A only
-log(p value)
Response to wounding
Tissue morphogenesis
Epithelial cell differentiation
Regulation of cell migration
Myeloid leukocyte differentiation
siGATA6 only
-log(p value)
Regulated exocytosis
Glycoprotein metabolic process
Regulation of hormone levels
Lipid biosynthetic process
Carbohydrate metabolic process
siHNF4A + siGATA6 only
-log(p value)
T cell differentiation
Extracellular matrix organization
Embryonic pattern specification
Bile acid and bile salt transport
Regulation of protein localization to plasma membrane

FIG. S6

Figure S6. HNF4A and GATA6 regulated target genes. (A and C) Immunoblots of HNF4A (A) and GATA6 (C) expression following 72 hours of transfection with either siNT (non-targeting control), siHNF4A (A) or siGATA6 (C). Tubulin expression was detected as a loading control. (B and D) RNA level expression measured by RT-qPCR analysis of *HNF4A* and *CLRN3* expression after 72 hours of siHNF4A transfection (B) or *GATA6* and *CLDN18* after 72 hours of siGATA6 transfection (D). Expression levels from RNA-seq data of *HNF4A* and *GATA6* following siRNA knockdown of the reciprocal factor shown on the right. Expression in the presence of siNT (non-targeting control) is taken as 1 in all cases. ** represents $p < 0.01$, *** represents $p < 0.001$ (n=3). (E) Correlation plots of replicate RNA-seq data from OE19 cells treated with siNT, siHNF4A, siGATA6 or both siHNF4A+siGATA6 with Pearson's correlation stated. (F) GO term analysis of differentially downregulated genes following siHNF4A, siGATA6 or siHNF4A+siGATA6 treatment. The genes included in the analysis direct targets of the respective transcription factors and are those uniquely downregulated by either HNF4A ("siHNF4A only") or GATA6 (siGATA6 only") depletion (and not also reciprocally regulated by the other factor) or genes that are upregulated either when both or either HNF4A or GATA6 are depleted or only when both factors are depleted ("siHNF4A and siGATA6"). The top 5 most significant terms are shown.

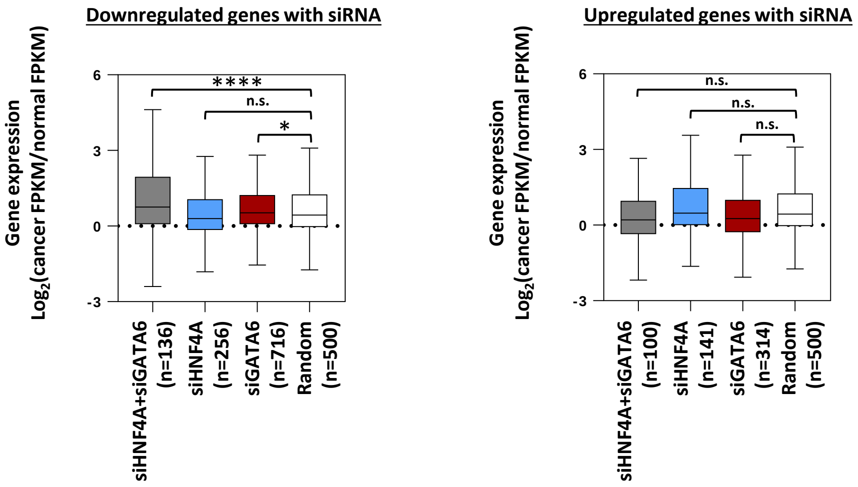

Figure S7. The expression of direct HNF4A-GATA6 target genes in oesophageal tissues. Boxplot of changes in gene expression in OAC tissue (shown as cancer/normal FPKM $\log_2$ fold change) for genes downregulated (A) and upregulated (B) by siRNA against HNF4A (blue), GATA6 (red) or both (black)(TCGA-IC-A6RE-11A/01A data; The Cancer Genome Atlas Research Network, 2017). Whiskers represent 1.5 x IQR. A random (500 randomly selected RefSeq transcripts) dataset is represented in white. **** represents $p<0.0001$, * represents $p<0.05$.

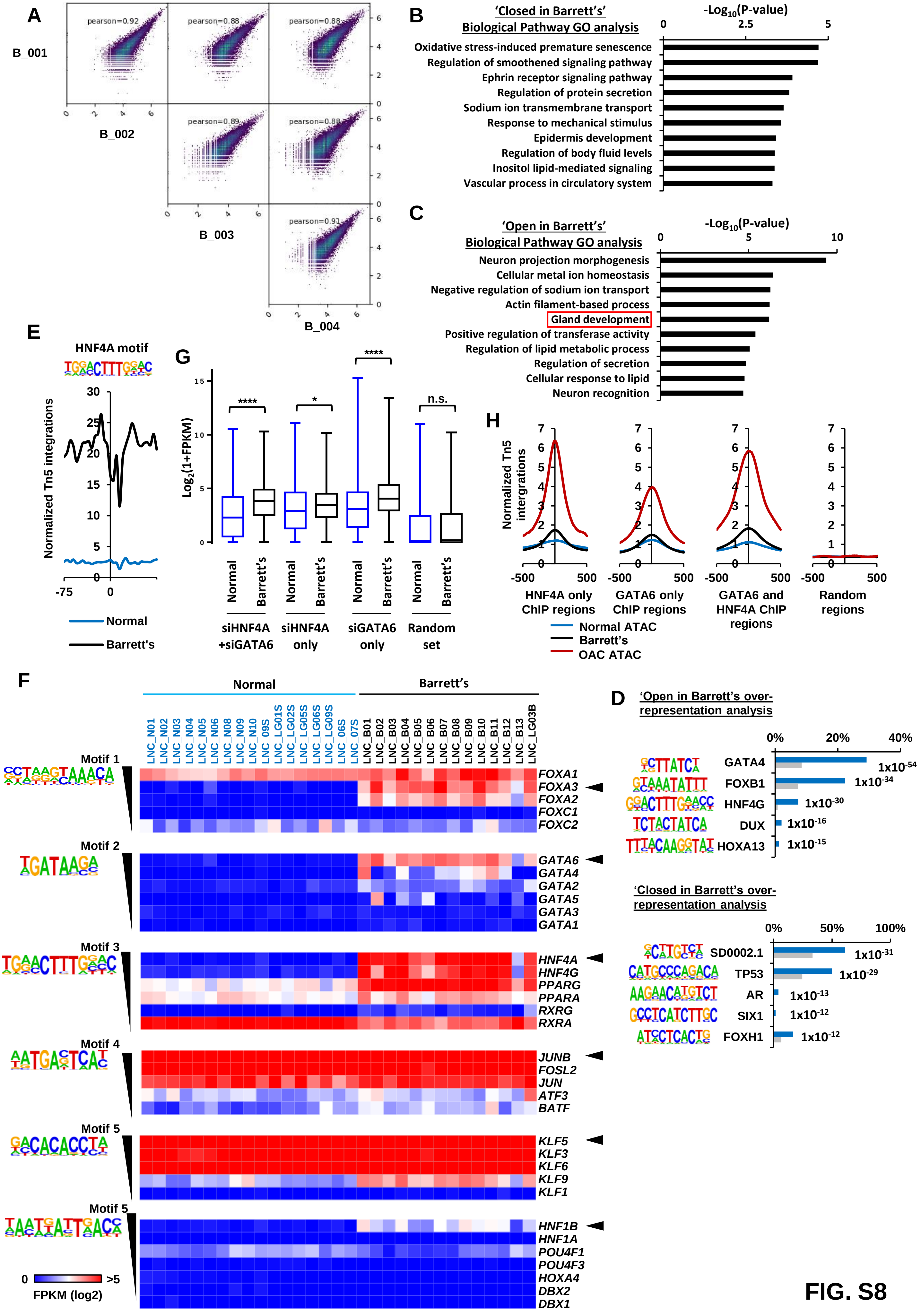
A
B_001
B_002
B_003
B_004
pearson=0.92
pearson=0.88
pearson=0.88
pearson=0.89
pearson=0.88
pearson=0.91
B
'Closed in Barrett's'
Biological Pathway GO analysis
-Log10(P-value)
Oxidative stress-induced premature senescence
Regulation of smoothened signaling pathway
Ephrin receptor signaling pathway
Regulation of protein secretion
Sodium ion transmembrane transport
Response to mechanical stimulus
Epidermis development
Regulation of body fluid levels
Inositol lipid-mediated signaling
Vascular process in circulatory system
C
'Open in Barrett's'
Biological Pathway GO analysis
-Log10(P-value)
Neuron projection morphogenesis
Cellular metal ion homeostasis
Negative regulation of sodium ion transport
Actin filament-based process
Gland development
Positive regulation of transferase activity
Regulation of lipid metabolic process
Regulation of secretion
Cellular response to lipid
Neuron recognition
E
HNF4A motif
Normalized Tn5 integrations
Normal
Barrett's
G
Log2(1+FPKM)
****
*
****
n.s.
Normal
Barrett's
siHNF4A +siGATA6
siHNF4A only
siGATA6 only
Random set
H
Normalized Tn5 intergrations
HNF4A only ChIP regions
GATA6 only ChIP regions
GATA6 and HNF4A ChIP regions
Random regions
Normal ATAC
Barrett's
OAC ATAC
F
Normal
Barrett's
Motif 1
Motif 2
Motif 3
Motif 4
Motif 5
Motif 5
FOXA1
FOXA3
FOXA2
FOXC1
FOXC2
GATA6
GATA4
GATA2
GATA5
GATA3
GATA1
HNF4A
HNF4G
PPARG
PPARA
RXRG
RXRA
JUNB
FOSL2
JUN
ATF3
BATF
KLF5
KLF3
KLF6
KLF9
KLF1
HNF1B
HNF1A
POU4F1
POU4F3
HOXA4
DBX2
DBX1
FPKM (log2)
D
'Open in Barrett's over-representation analysis
GATA4 1x10-54
FOXB1 1x10-34
HNF4G 1x10-30
DUX 1x10-16
HOXA13 1x10-15
'Closed in Barrett's over-representation analysis
SD0002.1 1x10-31
TP53 1x10-29
AR 1x10-13
SIX1 1x10-12
FOXH1 1x10-12

FIG. S8

Figure S8. Open chromatin profiling and gene expression in Barrett's tissue. (A) Correlation plots of ATAC-seq data from biopsies non-dysplastic Barrett's tissues (B_001 to B_004) with Pearson's correlation stated. (B and C) GO term analysis showing the top enriched biological pathways for genes associated with regions "closed in Barrett's" (B) or "open in Barrett's" (C). The top 10 most significantly enriched categories are shown. (D) Top five DNA motifs derived from de novo motif discovery (using total accessible regions in Barrett's as background rather than whole genome) and their associated transcription factor that are enriched in 'open in Barrett's' (top) or 'closed in Barrett's' (bottom) non-promoter regions. The frequency of motif occurrence is shown and the motifs are sorted by p-value. (E) Footprinting of ATAC-seq data from normal and Barrett's tissue on 150 bp regions surrounding the HNF4A transcription factor motif found in 'Open in Barrett's' regions. Normalised Tn5 integrations are plotted across each of the regions relative to the centre of the binding motif. Data from Barrett's tissue are shown in black and normal tissue in blue. (F) Heatmaps of log_2(1+FPKM) values of transcription factors with enriched DNA binding motifs in "open in Barrett's" regions in RNAseq data from normal or Barrett's tissue (Maag et al., 2017). Heatmaps are sorted by highest average fold increase expression in Barrett's (indicated by a triangle) and the highest expressed transcription factor in each case is indicated by an arrow. (G) Boxplot of log_2 (1+FPKM) values of the expression of genes positively regulated by either HNF4A, GATA6 or both transcription factors in either normal or Barrett's tissue (Maag et al., 2017). **** represents $p<0.0001$, * represents $p<0.05$. Median values are indicated by the horizontal lines. (H) Tag density plots of normalised ATAC-seq signal from merged normal (blue), Barrett's (black) and OAC (red) tissue from regions bound by HNF4A only, GATA6 only, both factors and random regions as a control.

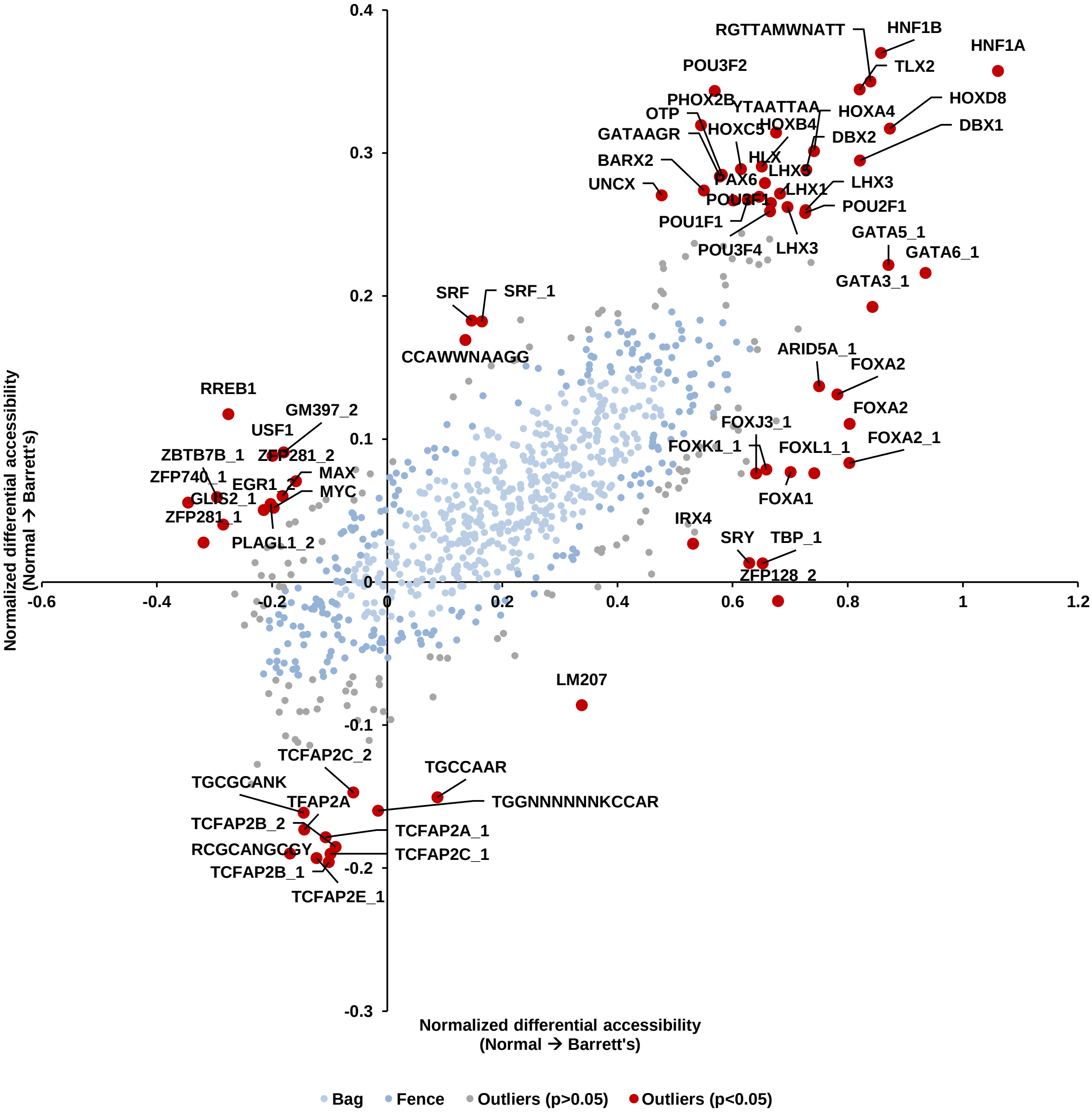

Figure S9. BaGFoot analysis of open chromatin regions in Barrett's tissue. Scatter bag plot of differential chromatin accessibility (x-axis) and footprinting (y-axis) depth around human transcription factor binding motifs in normal and non-dysplastic Barrett's tissue. Significant outliers (p<0.05) are represented in red. This is identical to Fig. 4D but with full labelling of the motifs included.

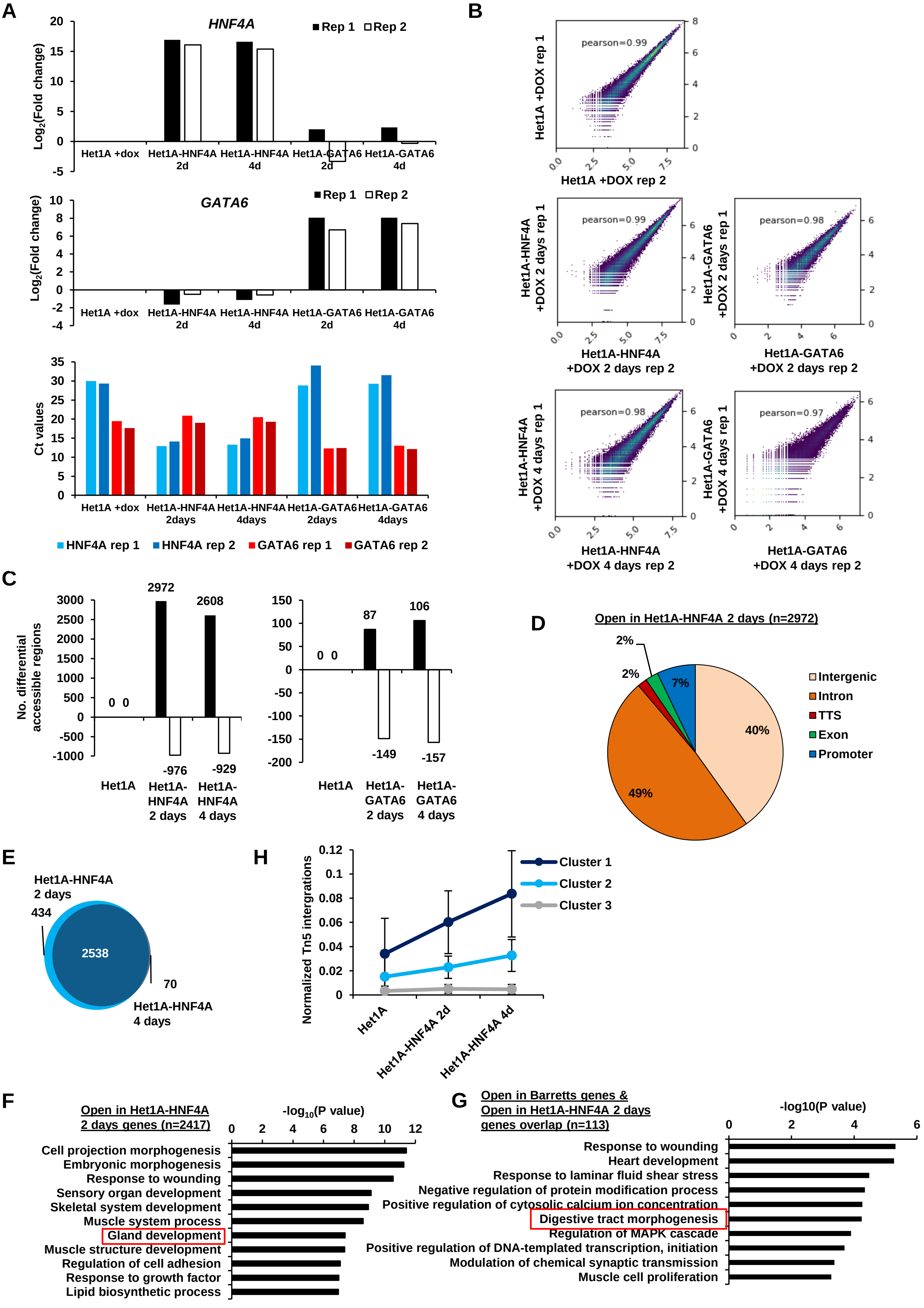

A
HNF4A
Rep 1 Rep 2
Log2(Fold change)
Het1A +dox
Het1A-HNF4A 2d
Het1A-HNF4A 4d
Het1A-GATA6 2d
Het1A-GATA6 4d
GATA6
Ct values
HNF4A rep 1 HNF4A rep 2 GATA6 rep 1 GATA6 rep 2
B
pearson=0.99
Het1A +DOX rep 1
Het1A +DOX rep 2
pearson=0.99
Het1A-HNF4A +DOX 2 days rep 1
Het1A-HNF4A +DOX 2 days rep 2
pearson=0.98
Het1A-GATA6 +DOX 2 days rep 1
Het1A-GATA6 +DOX 2 days rep 2
pearson=0.98
Het1A-HNF4A +DOX 4 days rep 1
Het1A-HNF4A +DOX 4 days rep 2
pearson=0.97
Het1A-GATA6 +DOX 4 days rep 1
Het1A-GATA6 +DOX 4 days rep 2
C
No. differential accessible regions
2972
2608
-976
-929
87
106
-149
-157
Het1A
Het1A-HNF4A 2 days
Het1A-HNF4A 4 days
Het1A-GATA6 2 days
Het1A-GATA6 4 days
D
Open in Het1A-HNF4A 2 days (n=2972)
40%
49%
7%
2%
2%
Intergenic
Intron
TTS
Exon
Promoter
E
Het1A-HNF4A 2 days
434
2538
70
Het1A-HNF4A 4 days
H
Normalized Tn5 intergrations
Cluster 1
Cluster 2
Cluster 3
Het1A
Het1A-HNF4A 2d
Het1A-HNF4A 4d
F
Open in Het1A-HNF4A 2 days genes (n=2417)
-log10(P value)
Cell projection morphogenesis
Embryonic morphogenesis
Response to wounding
Sensory organ development
Skeletal system development
Muscle system process
Gland development
Muscle structure development
Regulation of cell adhesion
Response to growth factor
Lipid biosynthetic process
G
Open in Barretts genes & Open in Het1A-HNF4A 2 days genes overlap (n=113)
-log10(P value)
Response to wounding
Heart development
Response to laminar fluid shear stress
Negative regulation of protein modification process
Positive regulation of cytosolic calcium ion concentration
Digestive tract morphogenesis
Regulation of MAPK cascade
Positive regulation of DNA-templated transcription, initiation
Modulation of chemical synaptic transmission
Muscle cell proliferation

FIG. S10

Figure S10. Open chromatin profiling of Het1A with overexpression of HNF4A or GATA6. (A) qRT-PCR of Het1A cells (treated with doxycline (dox) for four days), or Het1A-HNF4A and Het1A-GATA6 cells treated with doxycline (dox) for two and four days (d) of induction, using primers for *HNF4A* (top) and *GATA6* (middle). Ct values of samples are also shown (bottom). (B) Correlation plots of ATAC-seq replicates of Het1A cells (+dox), Het1A-HNF4A and Het1A-GATA6 cells (two and four days of induction) with Pearson's correlation stated. (C) Number of differentially accessible regions (linear 5 fold difference) with induction of HNF4A or GATA6 after two or four days dox treatment. (D) Pie chart of the genomic distribution of differentially accessible regions in Het1A-HNF4A after 2 days of dox treatment. Promoter is defined as -2.5 kb to +0.5 kb relative to the TSS. (E) Overlap of differentially more accessible regions between Het1A-HNF4A 2d and Het1A-HNF4A 4d cells with numbers indicated in each quadrant. (F) GO term analysis showing the top enriched biological pathways for genes associated with regions showing increased accessibility following induction of HNF4A expression in Het1A cells. The top 10 most significantly enriched categories are shown. (G) GO term analysis showing the top enriched biological pathway for genes associated with regions showing increased accessibility following induction of HNF4A expression and increased accessibility in Barrett's compared to normal tissue. (H) Average normalised ATAC-seq signal from Het1A or Het1A-HNF4A cells after 2 (2d) or 4 (4d) days induction for regions comprising the three *k*-means clusters in Fig. 5E).

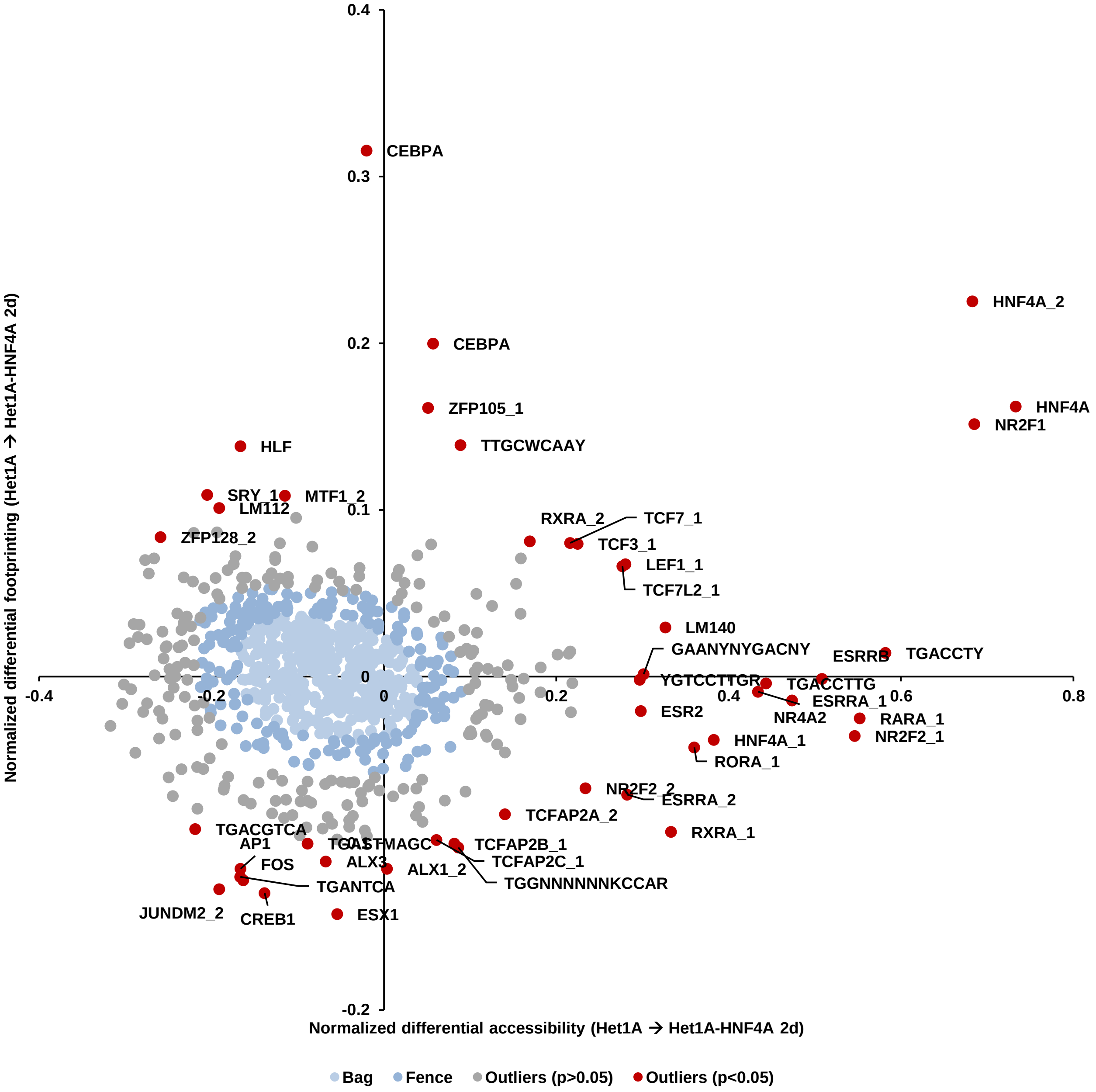

Figure S11. BaGFoot analysis of open chromatin regions in Het1A-HNF4A cells. Scatter bag plot of differential chromatin accessibility (x-axis) and footprinting (y-axis) depth around human transcription factor binding motifs in Het1A (+dox) and Het1A-HNF4A (2d) cells. Significant outliers (p<0.05) are represented in red. This is identical to Fig. 5C but with full labelling of the motifs included.

Supplemental Table S7. DNA motifs enriched in Barrett's-specific open chromatin regions.

Top ten motifs found by de novo motif discovery and their associated transcription factors that are enriched in 'open in Barrett's' (A) or 'closed in Barrett's' (B) non-promoter regions.

(A) Open in Barrett's; Non-promoter regions

Rank	Motif	P-value	log P-pvalue	% of Targets	% of Background	Best Match/Details
1	CCTAAGTAAACA	1.00E-95	-2.20E+02	40.32%	10.00%	FOXM1(Forkhead)/MCF7-FOXM1-ChIP-Seq(GSE72977)/Homer(0.940)
2	TGATAAGA	1.00E-70	-1.63E+02	26.54%	5.45%	Gata4(Zf)/Heart-Gata4-ChIP-Seq(GSE35151)/Homer(0.971)
3	TGAACTTTGGAC	1.00E-67	-1.54E+02	13.64%	1.14%	HNF4G/MA0484.1/Jaspar(0.961)
4	AATGACTCAT	1.00E-65	-1.52E+02	19.94%	3.11%	Atf3(bZIP)/GBM-ATF3-ChIP-Seq(GSE33912)/Homer(0.960)
5	GACACACCTA	1.00E-31	-7.34E+01	10.85%	1.91%	EKLF(Zf)/Erythrocyte-Klf1-ChIP-Seq(GSE20478)/Homer(0.839)
6	ATCATTAACC	1.00E-30	-7.12E+01	13.20%	2.97%	Nkx6.1(Homeobox)/Islet-Nkx6.1-ChIP-Seq(GSE40975)/Homer(0.787)
7	ACTTCCTGGT	1.00E-23	-5.51E+01	10.41%	2.38%	ELF3(ETS)/PDAC-ELF3-ChIP-Seq(GSE64557)/Homer(0.926)
8	AGGGTGGAGT	1.00E-19	-4.45E+01	10.56%	2.96%	KLF3(Zf)/MEF-Klf3-ChIP-Seq(GSE44748)/Homer(0.844)
9	CACTCCCT	1.00E-15	-3.54E+01	19.35%	9.24%	KLF5(Zf)/LoVo-KLF5-ChIP-Seq(GSE49402)/Homer(0.746)
10	TGATAGCTTG	1.00E-13	-3.16E+01	3.96%	0.58%	PH0169.1_Tgif1/Jaspar(0.659)

(B) Closed in Barrett's; Non-promoter regions

Rank	Motif	P-value	log P-pvalue	% of Targets	% of Background	Best Match/Details
1	CATGCCCAGACA	1.00E-46	-1.06E+02	58.35%	25.77%	p53(p53)/mES-cMyc-ChIP-Seq(GSE11431)/Homer(0.824)
2	CAAGCTTGTT	1.00E-36	-8.42E+01	58.58%	29.19%	Nr2e3/MA0164.1/Jaspar(0.810)
3	TGACTCAT	1.00E-35	-8.19E+01	30.89%	9.39%	BATF(bZIP)/Th17-BATF-ChIP-Seq(GSE39756)/Homer(0.980)
4	GTTACTGCATGT	1.00E-24	-5.62E+01	11.90%	1.92%	Ets1-distal(ETS)/CD4+-PolII-ChIP-Seq(Barski_et_al.)/Homer(0.616)
5	CCTGCCAA	1.00E-20	-4.81E+01	38.44%	18.86%	NFIA/MA0670.1/Jaspar(0.885)
6	AAGAACATGTCT	1.00E-17	-3.96E+01	4.81%	0.33%	AR-halfsite(NR)/LNCaP-AR-ChIP-Seq(GSE27824)/Homer(0.594)
7	TGCCTAAGGC	1.00E-16	-3.83E+01	13.27%	3.59%	TFAP2C(var.2)/MA0814.1/Jaspar(0.844)
8	GTTTTGCCCTGG	1.00E-15	-3.60E+01	2.06%	0.02%	ZNF416(Zf)/HEK293-ZNF416.GFP-ChIP-Seq(GSE58341)/Homer(0.612)
9	GCACACGC	1.00E-15	-3.47E+01	25.63%	11.69%	PB0130.1_Gm397_2/Jaspar(0.796)
10	CTTGCTAAGA	1.00E-13	-3.05E+01	3.43%	0.21%	PB0054.1_Rfx3_1/Jaspar(0.730)

Supplemental Table S9. DNA motifs enriched in chromatin regions that are more accessible upon HNF4A expression.

Rank	Motif	P-value	log P-pvalue	% of Targets	% of Background	Best Match/Details
1	TATGACTCAT	1e-330	-7.60E+02	28.30%	4.42%	Fra1(bZIP)/BT549-Fra1-ChIP-Seq(GSE46166)/Homer(0.983)
2	CAAAGTCCAA	1.00E-232	-5.36E+02	67.44%	34.51%	HNF4G/MA0484.1/Jaspar(0.899)
3	TAAGGAATGT	1.00E-94	-2.18E+02	21.55%	7.90%	TEAD3/MA0808.1/Jaspar(0.961)
4	TCTGCCCTGG	1.00E-64	-1.48E+02	28.60%	14.92%	PB0133.1_Hic1_2/Jaspar(0.810)
5	TTTGACCCTGGG	1.00E-53	-1.23E+02	27.25%	14.88%	EBF1(EBF)/Near-E2A-ChIP-Seq(GSE21512)/Homer(0.699)
6	TCTGTGGTTT	1.00E-43	-9.95E+01	12.99%	5.45%	RUNX(Runt)/HPC7-Runx1-ChIP-Seq(GSE22178)/Homer(0.959)
7	GTCCAATGAA	1.00E-27	-6.24E+01	7.84%	3.20%	PB0005.1_Bbx_1/Jaspar(0.722)
8	ATTATGAC	1.00E-18	-4.23E+01	4.05%	1.41%	PH0065.1_Hoxc10/Jaspar(0.621)
9	AGCTTTGACC	1.00E-17	-4.11E+01	3.08%	0.91%	MF0004.1_Nuclear_Receptor_class/Jaspar(0.750)
10	GGCAGTCCAA	1.00E-16	-3.91E+01	1.69%	0.30%	PB0134.1_Hnf4a_2/Jaspar(0.618)

Supplemental Table S10. Patient sample information.

Samples	Sex	Matched sample
N_003	M	
N_005	F	
N_006	M	
B_001	F	
B_002	M	
B_003	M	
B_004	F	
T_001	M	
T_002	M	
T_003	M	N_003
T_004	M	
T_005	F	N_005
T_006	M	N_006

Supplemental Table S11. PCR primers used in this study

ChIP-qPCR

Associated gene	Forward (5'-3')	ADS#	Reverse (5'-3')	ADS#
Control	AAGCCCCTCAACAGACTCAA	5857	CCTCATAGCTTGACCTTCGAG	5858
CLRN3_P1	TGGGAGAGGCAGGCTAACTA	5869	GGCCCGTCTCATTTTGAGTA	5870
CLRN3_P2	TGGGAGAGGCAGGCTAACTA	5871	GGCCCGTCTCATTTTGAGTA	5872
CLRN3_P3	CCGTGTGAAGGGACATCTTT	5873	GCACTCAGCAGGAGTGTCAG	5874
CLDN18_P1	GACCAATCTGCAGTGCCTTC	5901	GGGCTTGCCCTACTCCTTTA	5902
CLDN18_P2	CAAACAGAAACCAGCCCTCC	5903	GTGGGAAGTGTTAGGGCTGA	5904
CLDN18_P3	GAAAGCGAGTGTGAGGGAAG	6342	TGGTCACAGCATTTGGTGAT	6343

RT-qPCR

Gene	Forward (5'-3')	ADS#	Reverse (5'-3')	ADS#
RPLP0	ATTACACCTTCCCACTTGCT	5454	CAAAGAGACCAAATCCCATATCCT	5455
HNF4A	ATGTACTCCTGCAGATTTAGCC	5855	CCTCATAGCTTGACCTTCGAG	5856
GATA6	CTCTACAGCAAGATGAACGG	5430	TGGAGTTTCATGTAGAGTCCAC	5431
CLRN3	CAACCCTTACCAGACATTCC	5851	TATGAGCCAGAACGAGTATCC	5852
CLDN18	ATCATGTTCATTGTCTCAGGTC	5470	GGCTTTGTAGTTGGTTTCTTCTG	5471

Additional Supplemental Table legends

Table S1. Differentially accessible regions in OAC patient samples. (A) Coordinates of the top 50,000 genomic regions showing accessibility across normal and OAC samples. (B and C) Coordinates of regions showing >5 fold changes in accessibility when comparing the average signal in OAC tumour samples T_002, T_003, T_005, T_006 to normal tissue N_003, N_005 and N_006 among these 50,000 regions. Regions are split according to genomic locations in promoter regions (-2.5 kb to +0.5 kb relative to the TSS) (C) or non-promoter regions (B).

Table S2. DNA motifs enriched in OAC-specific open chromatin regions. Top ten motifs found by *de novo* motif discovery and their associated transcription factors that are enriched in 'open in cancer' (A) or 'closed in cancer' (B) promoter or non-promoter regions.

Table S3. HNF4A and GATA6 binding regions identified by ChIP-seq . Genomic regions identified by ChIP-seq for HNF4A (A) or GATA6 (B) binding.

Table S4. DNA motifs enriched in HNF4A and GATA6 ChIP-seq datsets. Top 25 motifs found by *de novo* motif discovery and their associated transcription factors that are enriched in HNF4A (A) or GATA6 (B) ChIP-seq datasets.

Table S5. Differentially regulated genes following transcription factor knockdown. Genes whose expression is deregulated by >1.3 fold (p-value<0.05) following either HNF4A (A and B), GATA6 (C and D) or both HNF4A and GATA6 (E and F) depletion. Data are split between genes up- and down-regulated. Only genes representing likely direct targets (i.e. associated with a nearby HNF4A and/or GATA6 peak) are shown.

Table S6. Differentially accessible regions in Barrett's patient samples. (A) Coordinates of the top 50,000 genomic regions showing accessibility across normal and Barrett's samples. (B and C) Coordinates of regions showing >5 fold increase or decrease in accessibility when comparing the average signal in Barrett's samples B_001-4 to normal tissue N_003, N_005 and N_006 among these 50,000 regions. Regions are split according to genomic locations in promoter regions (-2.5 kb to +0.5 kb relative to the TSS) (C) or non-promoter regions (B).

Table S8. Differentially accessible regions in Barrett's patient samples. (A) Coordinates of the top 50,000 genomic regions showing accessibility across Het1A and Het1A-HNF4A cells treated with doxycycline for 2 days. (B and C) Coordinates of regions showing >5 fold increase (B) or decrease (C) in accessibility when comparing the signal in Het1A compared to Het1A-HNF4A cells. (D) Coordinates of the top 50,000 genomic regions showing accessibility across Het1A and Het1A-GATA6 cells treated with doxycycline for 2 days. (E and F) Coordinates of regions showing >5 fold increase (E) or decrease (F) in accessibility when comparing the signal in Het1A compared to Het1A-GATA6 cells.
